# Identification of molecular subtypes based on PANoptosis-related genes and construction of a signature for predicting the prognosis and response to immunotherapy response in hepatocellular carcinoma

**DOI:** 10.3389/fimmu.2023.1218661

**Published:** 2023-08-18

**Authors:** Jinfeng Zhu, Qian Huang, Xingyu Peng, Chen Luo, Zitao Liu, Dongdong Liu, Huazhao Yuan, Rongfa Yuan, Xuexin Cheng

**Affiliations:** ^1^ Department of General Surgery, The Second Affiliated Hospital of Nanchang University, Nanchang, China; ^2^ Department of General Practice, Shanghai East Hospital, Tongji University School of Medicine, Shanghai, China; ^3^ Department of General Surgery, The First Affiliated Hospital of Nanchang University, Nanchang, China; ^4^ Department of General Surgery, Hukou County People’s Hospital, Jiujiang, China; ^5^ Department of General Surgery, Jiujiang Traditional Chinese Medicine Hospital, Jiujiang, China; ^6^ Biological Resource Center, The Second Affiliated Hospital of Nanchang University, Nanchang, China; ^7^ School of Public Health, Nanchang University, Nanchang, China; ^8^ Jiangxi Provincial Key Laboratory of Preventive Medicine, Nanchang University, Nanchang, China

**Keywords:** PANoptosis, hepatocellular carcinoma, molecular subtypes, signature, prognosis, immunotherapy response

## Abstract

**Background:**

Previous studies have demonstrated that PANoptosis is strongly correlated with cancer immunity and progression. This study aimed to develop a PANoptosis-related signature (PANRS) to explore its potential value in predicting the prognosis and immunotherapy response of hepatocellular carcinoma (HCC).

**Methods:**

Based on the expression of PANoptosis-related genes, three molecular subtypes were identified. To construct a signature, the differentially expressed genes between different molecular subtypes were subjected to multivariate least absolute shrinkage and selection operator Cox regression analyses. The risk scores of patients in the training set were calculated using the signature. The patients were classified into high-risk and low-risk groups based on the median risk scores. The predictive performance of the signature was evaluated using Kaplan-Meier plotter, receiving operating characteristic curves, nomogram, and calibration curve. The results were validated using external datasets. Additionally, the correlation of the signature with the immune landscape and drug sensitivity was examined. Furthermore, the effect of *LPCAT1* knockdown on HCC cell behavior was verified using *in vitro* experiments.

**Results:**

This study developed a PANRS. The risk score obtained by using the PANRS was an independent risk factor for the prognosis of patients with HCC and exhibited good prognostic predictive performance. The nomogram constructed based on the risk score and clinical information can accurately predicted the survival probability of patients with HCC. Patients with HCC in the high-risk groups have high immune scores and tend to generate an immunosuppressive microenvironment. They also exhibited a favorable response to immunotherapy, as evidenced by high tumor mutational burden, high immune checkpoint gene expression, high human leukocyte antigen gene expression, low tumor immune dysfunction and low exclusion scores. Additionally, the PANRS enabled the identification of 15 chemotherapeutic agents, including sorafenib, for patients with HCC with different risk levels, guiding clinical treatment. The signature gene *LPCAT1* was upregulated in HCC cell lines. *LPCAT1* knockdown markedly decreased HCC cell proliferation and migration.

**Conclusion:**

PANRS can accurately predict the prognosis and immunotherapy response of patients with HCC and consequently guide individualized treatment.

## 1 Introduction

In 2020, primary liver cancer accounted for more than 900,000 new cases and 800,000 cancer-related fatalities (1). Primary liver cancer predominantly manifests as hepatocellular carcinoma (HCC), which accounts for 75%–85% of all primary liver cases (2). HCC is a major threat to human health owing to its high prevalence and propensity for aggressive and often treatment-resistant disease progression. Currently, radical hepatectomy, liver transplantation, and local ablation are viable curative options for some patients with early-stage HCC (3). Patients with intermediate-stage or advanced-stage HCC can undergo locoregional therapy or systemic therapy (treatment with the multitargeted tyrosine kinase inhibitor sorafenib) (4). These therapeutic modalities have yielded promising results in achieving long-term disease control. However, the high drug resistance of HCC limits the efficacy of these therapeutic modalities (3, 5, 6). The efficacy of even promising immunotherapies is limited in some patients with HCC. Thus, the urgent task at hand is to identify a specific molecular signature that can effectively forecast the response of the target population to these treatments, ultimately enhancing their efficacy (7).

As the primary objective of cancer treatment is to eliminate the tumor cells, the induction of cancer cell death is a crucial therapeutic strategy for cancer (8). Programmed cell death (PCD) is an active mechanism to maintain body development and survival (9). The most well-studied PCD pathways are apoptosis, pyroptosis, and necroptosis (10). In apoptosis, dying cells are degraded into apoptotic bodies, which are removed by phagocytes to maintain homeostasis. Apoptosis is characterized by the maintenance of membrane integrity (11). Pyroptosis and necroptosis, which are relatively “violent” modes of PCD, are triggered by the activation of key pore-forming proteins. In pyroptosis and necroptosis, dying cells are ruptured, facilitating the release of potent inflammatory factors that protect the host against diverse external threats, including invading pathogens (12). Recent studies have identified a cell death process, called PANoptosis, in which apoptosis, pyroptosis, and necroptosis are simultaneously initiated in pathogen-infected cells (13). PANoptosis is regulated by a cytoplasmic multimeric protein complex called PANoptosome, which comprises key molecules required for the induction of pyroptosis, apoptosis, and necroptosis, promoting the pro-inflammatory cell death process (14–16). With the deepening of research, the role of PANoptosis in some tumors has also been confirmed. For example, Karki et al. demonstrated that ADAR promotes melanoma and colorectal cancer by suppressing ZBP1-mediated immune responses and PANoptosis (17). Recent studies have further suggested that the induction of PANoptosis is a potential therapeutic strategy for colon cancer. In particular, the pro-inflammatory cytokines tumor necrosis factor (TNF)-α and interferon (IFN)-γ exert growth-inhibitory effects on human colon cancer cells by triggering the onset of PANoptosis (18). The research team of Pan identified three different PANoptosis patterns in 1316 patients with gastric cancer and constructed a scoring system called PANscore (19). The survival and immune response of patients with gastric cancer were accurately predicted using PANscore. These findings improved our understanding of the function of PANoptosis in gastric cancer pathogenesis. However, the role of PANoptosis in the pathogenesis of HCC has not been previously reported. Analysis of the potential applicability of PANscore (19) or other PANoptosis scoring systems in HCC is a promising avenue for future research.

This study investigated the prognostic value of PANoptosis-related genes and developed a signature to predict the prognosis and immunotherapy response of patients with HCC. The clinical applicability of the signature was comprehensively evaluated and validated using an external dataset. Additionally, this study demonstrated that the signature was significantly correlated with the tumor immune microenvironment. Thus, these findings provide novel insights for developing individualized treatment plans for HCC patients with HCC and improving their clinical outcomes.

## 2 Methods

### 2.1 Data collection and processing

**Figure 1:**
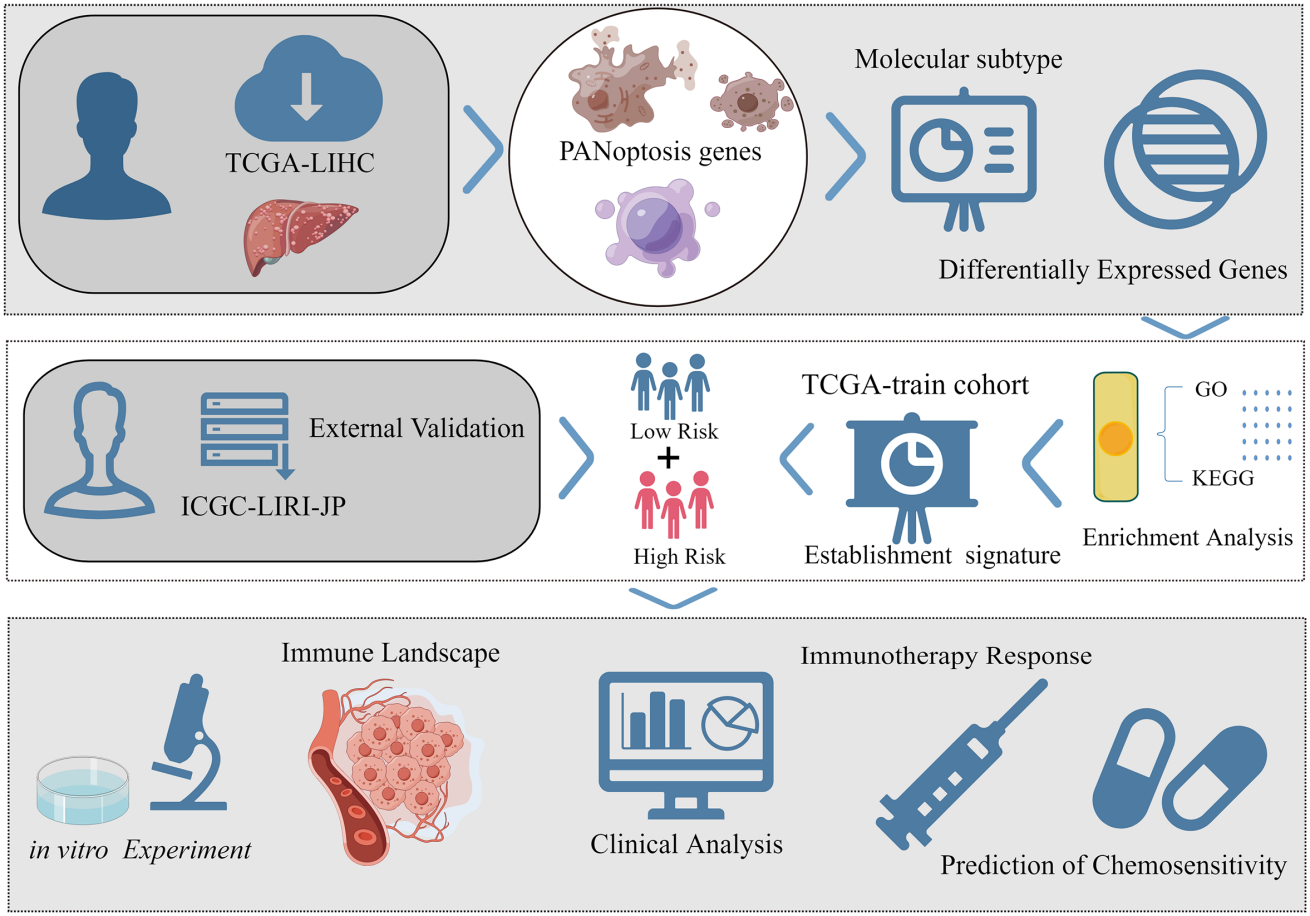
The brief flow chart of the study (By Figdraw, ID: AUYWT8d80f).

The workflow of this study is shown in **Figure 1**. The transcriptome data of 424 samples (50 non-cancerous samples and 374 tumor samples), the clinical data of 377 samples, and the mutation data of 368 samples of patients with liver hepatocellular carcinoma (LIHC) were downloaded from The Cancer Genome Atlas (TCGA) database. Additionally, the transcriptome data of 445 samples (202 non-cancerous samples and 243 tumor samples) and the clinical data of 260 samples in the International Cancer Genome Consortium-Liver cancer-Riken-Japan (ICGC-LIRI-JP) cohort were obtained from the ICGC database. Healthy samples and any samples with an unclear survival status or a survival time of < 30 days were excluded. After matching the transcriptome data from both databases with eligible survival data, 343 samples from TCGA-LIHC cohort were used for modeling and internal validation. Meanwhile, 230 samples of the ICGC-LIRI-JP cohort were used for external validation. Next, this study obtained a PANoptosis-related gene set comprising 25 pyroptosis-related genes, 8 necroptosis-related genes, and 32 apoptosis-related genes (**Table S1**) from previous studies (19). To objectively evaluate the differential expression levels of PANoptosis-related genes between liver cancer tissue and non-cancerous liver tissues, the transcriptome data of 110 normal liver tissue were extracted from the University of California Santa Cruz Xena’s Genotype-Tissue Expression(GTEx) project to narrow the gap in sample size between the two tissues.

### 2.2 Identification of molecular subtypes of PANoptosis

The PANoptosis molecular subtypes were identified using the consensus clustering algorithm based on the expression of the PANoptosis-related genes in TCGA-LIHC datasets. The clustering process was set to 50 iterations, and 80% of the sample data in each iteration were subsampled to identify a stable and reliable typing. To assess the validity of the classification, visualization was performed using principal component analysis (PCA). The single-sample gene set enrichment analysis (ssGSEA) was used to quantify the abundance of immune cell infiltration (on a scale of 0 to 1). Additionally, the levels of infiltrating immune cells in the three molecular subtypes were compared using the Kruskal-Wallis test. Gene set variation analysis (GSVA) was used to assess the biological functions associated with the molecular subtypes of PANoptosis. The screening criteria to identify the significantly enriched pathway between each two subtypes were as follows: |log fold-change (logFC)| > 0.1 and P (false discovery rate (FDR)) < 0.05. Differentially expressed genes (DEGs) between the molecular subtypes of PANoptosis were identified based on the following criteria: [logFC| ≥ 0.585 and FDR < 0.05.To understand the potential function, DEGs were subjected to Kyoto Encyclopedia of Genes and Genomes (KEGG) and Gene ontology (GO) enrichment analyses (the criterion for significant enrichment was q value < 0.05).

### 2.3 Establishment and identification of PANoptosis-related signature in TCGA cohort

Univariate Cox regression analyses were performed with the expression levels of DEGs in 343 samples of TCGA-LIHC cohort as independent variables and survival time and status as dependent variables *(p* < 0.05 indicates that DEGs are associated with prognosis). TCGA-LIHC cohort combined with prognostic-related DEGs was randomly split into training and test cohorts in the ratio 1:1. The least absolute shrinkage and selection operator (LASSO) algorithm (when partial likelihood deviance is at its lowest) and multivariate Cox regression analysis were used to further filter prognosis-related DEGs in the training cohort. Finally, a PANRS was obtained. The LASSO method is a compression estimator that can generate finer models by constructing penalty functions. The advantages of the LASSO method include compressing the number of coefficients and reducing multicollinearity problems in regression analysis (20). This study developed a new risk score for patients with HCC based on a gene signature. The risk score was calculated as the sum of the weighted expression of individual genes as follows: risk score = Σ (expression (Gene_n_) × coefficient (Gene_n_)) (where expression (Gene_n_) represents the expression of a specific gene and coefficient (Gene_n_) is its corresponding coefficient). The median risk score of the training cohort was used as the optimal threshold to classify TCGA-LIHC, training, test, and ICGC-LIRI-JP cohorts into high-risk and low-risk groups. To evaluate the prognostic accuracy of the signature, receiver operating characteristic (ROC) curve analysis was performed, and the C-index was calculated. The potential clinical relevance of this signature was determined using a clinical applicability analysis. Additionally, a nomogram was developed to further evaluate the accuracy of the signature using a calibration curve.

### 2.4 Prediction of immune landscape, immunotherapy response, and chemosensitivity

The cell-type identification by estimating relative subsets of RNA transcripts algorithm was used to obtain the abundance scores of 22 infiltrating immune cells for each TCGA-LIHC sample (total score was 1; *p* < 0.05 indicates that the accuracy of the levels of infiltrating immune cells obtained in a sample is good). The correlation of the risk scores or risk groups with the abundance of accurate immune cell infiltration was determined using Spearman’s or Wilcoxon tests, respectively. The stromal and immune scores, as well as the combined scores of the two components, in the tumor immune microenvironment were determined using the Estimation of STromal and Immune cells in MAlignant Tumours using Expression data (ESTIMATE) algorithms. The scores of the high-risk and low-risk groups were compared using the Wilcoxon test. Tumor immune dysfunction and exclusion (TIDE), exclusion, and dysfunction, and microsatellite instability (MSI) scores were used to predict the response of HCC to immunotherapy using the TIDE website (http://tide.dfci.harvard.edu/). Gene expression and clinical information of the immunotherapy cohort are stored in a public database (IMvigor210) (21). The effect of the signature gene lysophosphatidylcholine acyltransferase 1(*LPCAT1*) on the prognosis of the bladder cancer immunotherapy cohort was evaluated using the Comprehensive Analysis on Multi-Omics of Immunotherapy in Pan-cancer online website (http://camoip.net/). Tumor mutation burden (TMB) can predict immune checkpoint inhibitor (ICI) treatment response as it contributes to the production of neoantigens that activate anti-tumor immune responses (22). Additionally, the neoantigen, as a tumor-specific mutant peptide, is presented only by major histocompatibility complex (MHC) molecules (23). MHC-1 was reported to predict durable ICI response (24). Therefore, this study examined the expression of human leukocyte antigen (HLA) molecules in different risk groups. The variations in each parameter among different groups were analyzed using Spearman’s correlation analysis. The half-maximal inhibitory concentration (IC50) of drugs was predicted using the R package “pRRophetic.”

### 2.5 Cell culture and transfection

HCC cell lines (HCCLM3 (BNCC338460), MHCC97-H (BNCC359345), and HepG2 (CL-0103)) and hepatic epithelial cells (THLE-2 (BFN60808733)) were obtained from BeNa Culture Collection (Suzhou, China), Procell (Wuhan, China) and Qingqi Biotechnology Development Co., Ltd (Shanghai, China). These cell lines were cultured in complete medium of Dulbecco’s modified Eagle’s medium. The HepG2 and HCCLM3 cells were transfected in 6-well plates (NEST Biotechnology, Wuxi, China) using Lipofectamine 2000 (Invitrogen), following the manufacturer’s instructions. The corresponding small-interfering RNA (siRNA) sequences are listed in **Table S2**. HCC cells were subjected to pyroptosis induction using LPS (1 μg/mL) and ATP (100 μM) as in previous studies (25).

### 2.6 Quantitative real-time PCR (qRT-PCR) and western blotting

Total RNA was isolated from cells using the Steady Pure Quick RNA extraction kit (Accurate Biotechnology, AG21023). The isolated RNA was reverse-transcribed into complementary DNA using the Evo Moloney-murine leukemia virus reverse transcription premix kit (Accurate Biotechnology, AG11728). qRT-PCR was performed in a LightCycler (Roche, Germany) with 2× Universal SYBR Green Fast qPCR Mix (Abclonal, RK21203). The mRNA expression of *LPCAT1* was calculated using the 2^−ΔΔCt^ method. The primer sequences are shown in **Table S2.** Western blotting was performed a western blot as described previously (26). The following antibodies were used in western blotting analysis: anti-*LPCAT1* antibody (1:500, Proteintech, 16112-1-AP), anti-*CASP1* (1:6000, Proteintech, 22915-1-AP), anti-*CASP3* (1:500, ABclonal, A2156), anti-*CASP7* (1:600, Proteintech, 27155-1-AP), anti-*CASP8* (1:500, ABclonal, A0215), anti-*GSDME* (1:5000, Proteintech, 13075-1-AP), anti-cleaved *GSDME* antibody(1:500, ABclonal, A23072), anti-*GSDMD* (Full Length+N terminal, 1:500, ABclonal, A20197), anti-*MLKL* (1:500, ABclonal, A21894), anti-phospho-*MLKL* antibody(1:50, ABclonal, A1244), and anti-*GAPDH* antibody (1:10000, Proteintech, 60004-1-Ig).

### 2.7 *In vitro* experiments

Cell proliferation was evaluated using ethynyl-2-deoxyuridine (EdU) staining. Cells from the experimental and control groups were seeded at a density of 20,000 cells per well (100 µL) in a 96-well plate. The EdU-positive cell experiments were conducted using the EdU Apollo 488 kit (RiboBio, C10310-1). EdU‐positive cells rate (%) was calculated as follows: EdU-positive cell rate (%) = [number of EdU‐positive cells (green)/number of Hoechst‐33342-positive cells (blue)] × 100%.

Cell proliferation was examined using the cell counting kit-8 (CCK-8). Cells from the experimental and control groups were seeded in a 96-well plate at a density of 4000 cells (100 µL) per well. The proliferation of HepG2 and HCCLM3 cells at 0, 24, 48, and 72 h post-seeding was determined using CCK-8 reagent (GK10001, GLPBIO, Montclair, USA). The optical density at 450 nm of the reaction mixture was determined using an automatic microplate reader.

The migration ability of HCC cells was assessed using the wound-healing assay. Cells from the experimental group and the control groups were seeded in a 6-well culture plate. When the confluency of cells reached approximately 90%, a scratch was introduced in the monolayer using a 200-µL micropipette tip. The wound closure rate, the following formula was quantified as follows: Wound closure rate (%) = [(original wound area − unhealed wound area)/original wound area] × 100%.

Cell migration was examined using the Transwell assay. HCC cells were trypsinized, and the cell suspension was centrifuged at 1000 rpm for 5 min. The cell pellet was resuspended in phosphate-buffered saline and centrifuged. Next, the cell pellet was resuspended in a serum-free medium. The appropriate amount of cell suspension (300 μL) was inoculated into the Transwell chamber (20,000 cells per well for HCCLM3 and 100,000 cells per well for HepG2) and cultured for 24 h. Non-migrated cells were removed by gently scraping with a cotton swab. The migrated cells were immobilized by using 4% paraformaldehyde, stained with 0.2% crystal violet, imaged, and counted using ImageJ software.

### 2.8 Statistical analysis

Statistical analysis was performed using R software v4.1.3 and GraphPad Prism. Means between two groups were compared using the Wilcoxon test, while those between three groups were compared using the Kruskal test. The Kaplan-Meir method was used to generate survival curves, which were compared using the log-rank test. *In vitro* experiments were performed in triplicate, and the data were analyzed using Student’s t-test. Differences were considered significant at p < 0.05.

## 3 Results

### 3.1 Expression of PANoptosis-related genes and their prognostic relevance in HCC

**Figure 2:**
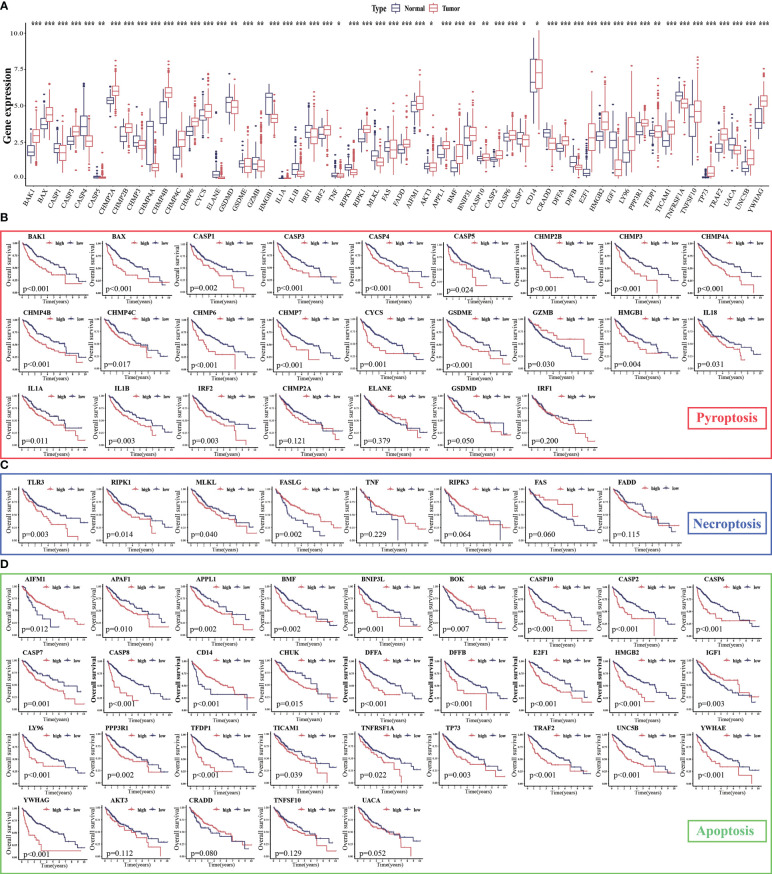
Expression levels of PANoptosis-related genes in hepatocellular carcinoma (HCC) and their correlation with survival. **(A)** The mRNA expression levels of PANoptosis-related genes in HCC and non-cancerous/para-cancerous samples were analyzed in The Cancer Genome Atlas (TCGA) and Genotype-Tissue Expression (GTEx) datasets; **(B–D)** Differential survival of patients in the high-expression and low-expression groups. ^*^ *p* < 0.05, ^**^ *p* < 0.01, and ^***^ *p* < 0.001.

The expression levels of 86.15% (56/65) of the PANoptosis-related genes were significantly different between HCC (n = 374) and non-cancerous tissues (n = 160). Of these, 33 PANoptosis-related genes were upregulated in HCC samples (11 pyroptosis-related genes, 2 necroptosis-related genes, and 20 apoptosis-related genes), whereas 23 PANoptosis-related genes were downregulated (12 pyroptosis-related genes, 4 necroptosis-related genes, and 7 apoptosis-related genes; **Figure 2A**). Additionally, the expression levels of most PANoptosis-related genes (84.54% (53/65); 21 pyroptosis-related genes, 4 necroptosis-related genes, and 28 apoptosis-related genes) were correlated with the overall survival(OS) in patients with HCC (**Figures 2B–D**). In general, the high-expression group exhibited a poor prognosis. These findings support further analysis of PANoptosis-related genes.

### 3.2 Identification of molecular subtypes based on PANoptosis-related genes

**Figure 3:**
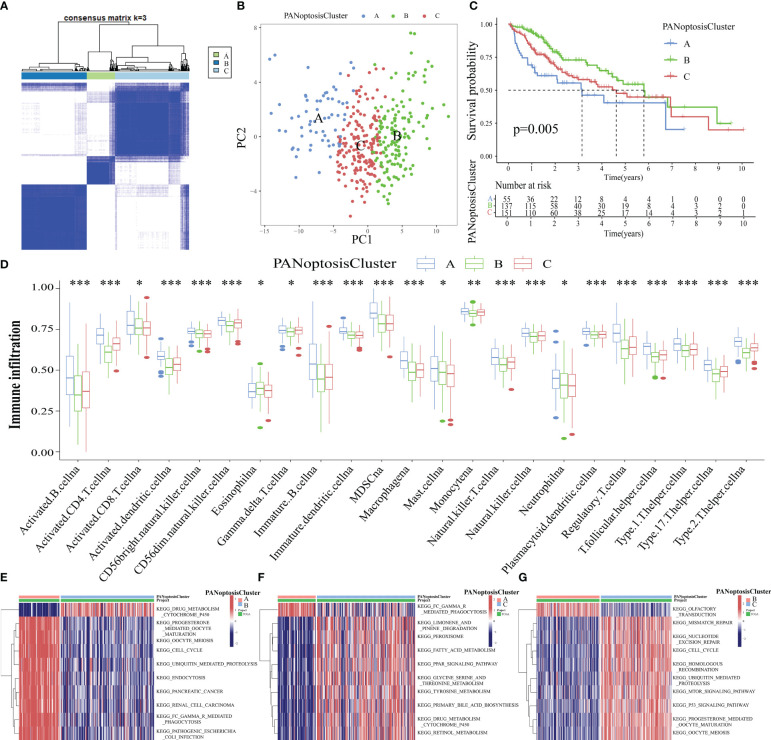
Identification of three molecular subtypes of PANoptosis. **(A)** Three PANoptosisClusters were identified using consensus clustering based on the expression levels of PANoptosis-related genes in The Cancer Genome Atlas-Liver Hepatocellular Carcinoma (TCGA-LIHC) cohort; **(B)** The three PANoptosisClusters were visually distinguished using principal component analysis (PCA); **(C)** Comparison of overall survival (OS) between the three PANoptosisClusters; **(D)** Differential immune cell infiltration status among the three PANoptosisClusters; **(E–G)** The differential biological function between the following pairs was examined using gene set variation analysis (GSVA): PANoptosisClusters A and B **(E)**, PANoptosisClusters A and C **(F)**, PANoptosisClusters B and C **(G)**. ^*^ *p* < 0.05, ^**^ *p* < 0.01, and ^***^ *p* < 0.001.

The intersection genes of TCGA-LIHC datasets and PANoptosis-related gene set were extracted and matched to the transcriptome data of TCGA-LIHC tumor samples to obtain the expression levels of 65 PANoptosis-related genes in 371 tumor samples. These 371 samples were divided into 3 subtypes, namely PANoptosisClusters A, B, and C, using consensus clustering (**Figure 3A**). The PCA plot provided an intuitive and clear depiction of the three distinct expression patterns in the PANoptosisClusters (**Figure 3B**). To further explore the effect of molecular subtypes of PANoptosis on the prognosis of HCC, the different subtypes were subjected to survival and immune infiltration analyses. The OS of patients with HCC significantly varied according to the molecular subtypes of PANoptosis. PANoptosisCluster B exhibited the best OS, followed by PANoptosisClusters C and A (**Figure 3C**). Additionally, patients in the PANoptosisCluster A exhibit a tumor microenvironment characterized by increased immune cell infiltration. Consistently, PANoptosisCluster B with the best OS exhibited the lowest infiltration of most immune cells. In addition to tumor-promoting immune cells (i.e. myeloid-derived suppressor cells and mast cells), the infiltrating immune cells included anti-tumor immune cells (CD8 (+) T cells and natural killer (NK) cells) and immune cells with both pro-tumor and anti-tumor effects (CD4 (+) T cells and dendritic cells) (**Figure 3D**). Recent single-cell transcriptome sequencing studies on HCC have confirmed that different immune cells express different PANoptosis-related genes (*CRADD* is upregulated in tumor-specific infiltrating regulatory T cells; *TNF* is upregulated in cytotoxic FGFBP2+ double‐positive T cells). The same PANoptosis-related gene can be upregulated in multiple immune cells (e.g. NK cells and GNLY+ T cells (cytotoxic CD4T cells) exhibit upregulated expression of *GZMB*) (27–29). This suggests that immune cells may express several PANoptosis-related genes and that the levels of infiltrating immune cells can indicate the expression level of PANoptosis-related genes to a certain extent. This can explain the high abundance of immune cell infiltration in PANoptosisCluster A with higher expression levels, while the low abundance of immune cell infiltration in PANoptosisCluster B with lower expression levels.

Next, the biological functions of different PANoptosis molecular subtypes were examined using GSVA. The top 10 enriched pathways between PANoptosisClusters A and B, between PANoptosisClusters A and C, and between PANoptosisClusters B and C are shown in **Figures 3E–G**, respectively. The enrichment of progesterone-mediated oocyte maturation and oocyte meiosis in PANoptosisCluster A was higher than that in PANoptosisCluster B. Additionally, the enrichment of drug metabolism cytochrome p450 in PANoptosisClusters B and C was higher than that in PANoptosisCluster A. PANoptosisCluster B was enriched in olfactory transduction, while PANoptosisCluster C was enriched in mTOR and other signaling pathways. Progesterone-mediated oocyte maturation and oocyte meiosis, which were significantly enriched in PANoptosisCluster A, are closely correlated to HCC progression (30, 31). However, cytochrome P450, an important biosynthetase in drug metabolism, enriched in PANoptosisClusters B and C can inhibit HCC growth by antagonizing HGF/MET signaling or AKT signaling (32, 33). These findings can explain the poor prognosis of PANoptosisCluster A. Additionally, in the olfactory transduction pathway, which was enriched in PANoptosisCluster B, OR1A2 is reported to suppress the proliferation of human HCC Huh-7 cells upon activation with (–)-citronellal (34). The mTOR signal transduction, which was significantly enriched in PANoptosisCluster C, can accelerate HCC progression upon PRIM1 activation (35). These results support that the OS of PANoptosisCluster B is higher than that of PANoptosisCluster C.

### 3.3 Construction of PANRS

**Figure 4:**
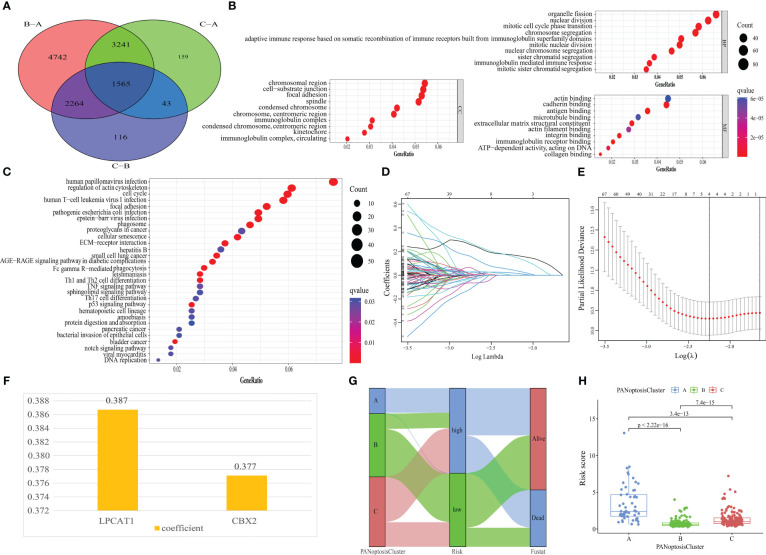
Establishment of PANoptosis-related signature (PANRS). **(A)** Screening of differentially expressed genes (DEGs) between the three PANoptosisClusters; **(B, C)** Gene Ontology (GO) **(B)** and Kyoto Encyclopedia of Genes and Genomes (KEGG) enrichment analyses **(C)** were performed to examine the biological function and related pathways in which the DEGs were enriched; **(D)** The trajectories of the coefficients of prognosis-related DEGs; **(E)** The smallest parameter graph to determine the number of included genes based on the cross-validation error; **(F)** Names of genes in PANRS and their corresponding risk coefficients; **(G)** The Sankey diagram shows how the molecular subtype of PANoptosis is quantified into a prognostic PANRS; **(H)** Distribution of risk scores among the PANoptosisClusters.

A PANRS was constructed to enable the application of these molecular subtypes for the clinical analysis of patients with HCC.Differential analysis of PANoptosisClusters A, B, and C revealed 1565 DEGs (**Figure 4A**). GO and KEGG functional enrichment analyses (**Figures 4B, C**) revealed that the main cellular component in which DEGs were enriched was the chromosomal region. Aberrations in chromosomal regions often lead to HCC cell proliferation, HCC exacerbation, and multiple drug resistance development (36–38). Additionally, the main biological process in which DEGs were enriched was organelle fission. The enhancement of organelle fission is reported to promote HCC metastasis (39, 40) and limit tumor immune surveillance by NK cells (41). Furthermore, the main molecular function in which DEGs were enriched was actin binding. This implies that DEGs can accelerate the progression of HCC by promoting actin binding (42, 43). The KEGG pathway in which DEGs were enriched was human papillomavirus (HPV) infection. Hepatitis B virus infection is a well-known adverse factor for HCC. However, the simultaneous infection of these cells with HPV 16 upregulates the transcriptional activity in HCC (44).

Next, DEGs were subjected to univariate Cox regression analysis that matched transcriptome data and survival information (n=343) and extracted 1155 DEGs associated with prognosis (**Table S3**). To facilitate subsequent internal cohort validation, TCGA-LIHC cohort (n = 343) was randomly divided into training and test cohorts before establishing the signature. The survival time, survival status, tumor grade and stage, and other important clinical indicators were not significantly different between the two groups (**Table 1**), indicating that the test cohort can be used as an internal validation cohort.

**Table 1:** Comparison of important clinical indicators in train cohort and test cohort.

Indicators	Train cohort	Test cohort	P value
Survival time(year)			0.82
<1	43 (25.00%)	39 (22.81%)	
>=5	21 (12.21%)	19 (11.11%)	
1-<5	108 (62.79%)	113 (66.08%)	
Survival state			
Alive	107 (62.21%)	113 (66.08%)	0.53
Deceased	65 (37.79%)	58 (33.92%)	
Age			
<=65	107 (62.21%)	109 (63.74%)	0.86
>65	65 (37.79%)	62 (36.26%)	
Gender			
Female	53 (30.81%)	57 (33.33%)	0.70
Male	119 (69.19%)	114 (66.67%)	
Grade			
G1-2	110 (63.95%)	104 (60.82%)	0.67
G3-4	60 (34.88%)	64 (37.43%)	
unknow	2 (1.16%)	3 (1.75%)	
Stage			
I-II	120 (69.77%)	118 (69.01%)	0.68
III-IV	39 (22.67%)	44 (25.73%)	
unknow	13 (7.56%)	9 (5.26%)	

As detecting a large number of genes in clinical practice is challenging, prognostic DEGs in the training cohort were subjected to LASSO Cox regression analysis to control the number of genes included in the signature. As shown in **Figure 4D**, the gene coefficient trajectories revealed that an increase in the penalty coefficient (Log λ) results in fewer genes included in the signature, as evidenced by a high proportion of genes with a coefficient of 0. To obtain a signature with an improved fitting effect, the four genes with the smallest cross-validation error in **Figure 4E** were selected and marked. These genes were subjected to multivariate Cox regression analysis. An optimal signature related to the molecular subtypes of PANoptosis, namely PANRS, was obtained. The PANRS risk score was calculated as follows: risk score = *LPCAT1* expression level × 0.387 + chromobox 2(*CBX2*) expression level × 0.377 (**Figure 4F**). The median risk score of 0.843 was the cut-off value for classifying the train, test, TCGA-LIHC, and ICGC-LIRI-JP cohorts into high-risk and low-risk groups. The Sankey diagram shows the brief process of predicting the prognosis of HCC after the molecular subtypes of PANoptosis were quantified as PANRS (**Figure 4G**). Additionally, comparative analysis of the distribution of risk scores in PANoptosisClusters revealed that PANoptosisCluster A had the highest risk score, followed by PANoptosisCluster C (**Figure 4H**) and PANoptosisCluster B. These results seem to explain the OS of different PANoptosisClusters. Thus, PANRS can well reflect the differential OS between PANoptosisClusters.

### 3.4 PANRS reliably predicts HCC prognosis in TCGA cohort

**Figure 5:**
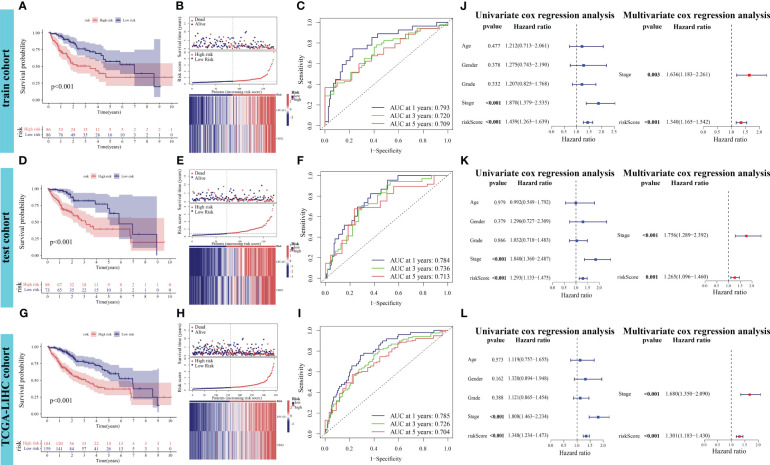
Verification of the prognostic performance of PANoptosis-related signature (PANRS) in The Cancer Genome Atlas-Liver Hepatocellular Carcinoma (TCGA-LIHC) cohort. **(A, B)** Differential overall survival (OS) and risk curve between the high-risk and low-risk groups in the training cohort; **(C)** In the training cohort, PANRS predicted the area under the curve (AUC) values for 1-year, 3-year, and 5-year OS of patients with hepatocellular carcinoma (HCC); **(D, E, G, H)** Differential OS **(D, G)** and risk curve **(E, H)** between the high-risk and low-risk groups in the test and TCGA-LIHC cohorts; **(F, I)** In the test **(F)** and TCGA-LIHC cohorts **(I)**, PANRS predicted the AUC values for 1-year, 3-year, and 5-year OS of patients with HCC; **(J–L)** Validation of the independent prognostic performance of PANRS in the training **(J)**, test **(K)**, and TCGA-LIHC cohorts **(L)**.

The differential OS between the high-risk and low-risk groups in the training cohort was analyzed. Patients in the high-risk group exhibited markedly decreased OS (*p* < 0.001, **Figure 5A**). The risk curve results indicated that patients in the high-risk group were associated with increased mortality rates and upregulated expression levels of the poor prognostic genes *LPCAT1* and *CBX2* (**Figure 5B**). In the training cohort, the performance of PANRS to predict the OS of patients with HCC was evaluated using time-dependent ROC curves. The area under the curve (AUC) values for predicting 1-year, 3-year, and 5-year OS were 0.793, 0.720, and 0.709, respectively (**Figure 5C**). Similar results were obtained in the test and TCGA-LIHC cohorts (**Figures 5D, E, G, H**). Additionally, the AUC (time-dependent ROC curve) values of PANRS in both test and TCGA-LIHC cohorts were > 0.7 (**Figures 5F, I**), indicating the high prognostic prediction performance of PANRS. Finally, independent prognostic analyses of risk scores in the three cohorts were performed to exclude the interference of other clinical factors. The risk scores were identified as independent prognostic factors for HCC (*p* < 0.01, **Figures 5J–L**).

**Figure 6:**
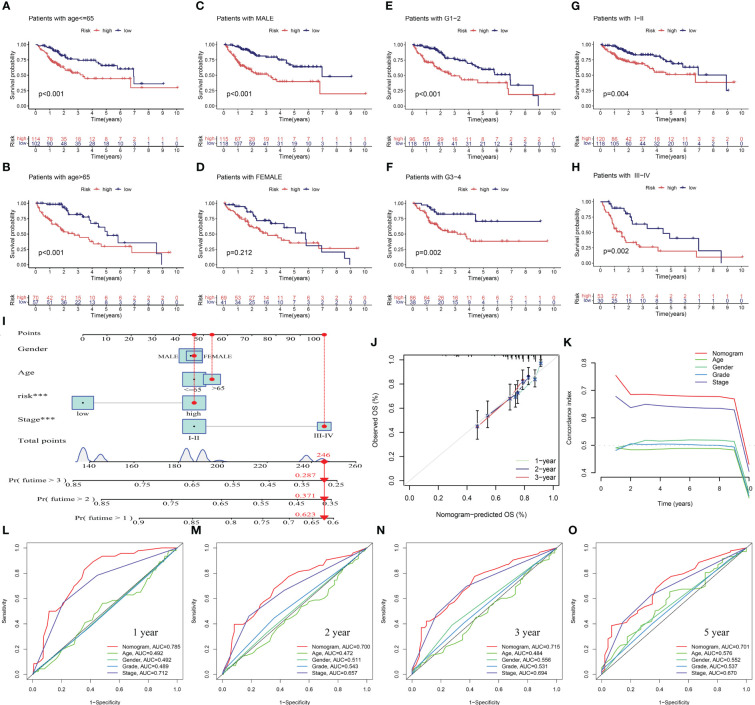
Evaluation of the potential clinical application value of PANoptosis-related signature (PANRS). **(A–H)** The clinical applicability of PANRS was evaluated by comparing the overall survival (OS) of the high-risk and low-risk groups according to age **(A, B)**, gender **(C, D)**, and tumor grades **(E, F)** and stages **(G, H)**; **(I)** A nomogram was constructed by combining the risk scores with age, sex, and tumor stage; **(J, K)** Calibration curve **(J)** and C-index **(K)** confirmed the high accuracy of the nomogram; **(L–O)** The ability of nomogram to predict 1-year **(L)**, 2-year **(M)**, 3-year **(N)**, and 5-year **(O)** OS in HCC was compared with other common clinical indicators through receiving operating characteristic (ROC) mapping.

To assess the potential clinical utility of PANRS, data of TCGA-LIHC cohort were subjected to an applicability analysis. When factors, such as age, sex (male), tumor grade, and tumor stage were considered, the OS of the high-risk group was significantly worse when compared with that of the low-risk group (all *p* < 0.01, **Figures 6A–H**). This suggests that PANRS can be widely used to predict the survival of HCC patients with HCC. To individually determine the probability of 1-year, 2-year, and 3-year survival rates for each patient, a nomogram was established by combining clinicopathological features and risk scores. As shown in **Figure 6I**, the comprehensive risk score of a patient was 246 points, and the 1-year, 2-year, and 3-year survival probabilities were predicted to be 62.3%, 37.1%, and 28.7%, respectively. The calibration curve in **Figure 6J** revealed a high level of consistency between the predicted 1-year, 2-year, and 3-year survival probabilities and actual survival rates. Moreover, the C-index and AUC values of the nomogram for predicting 1-year, 2-year, 3-year, and 5-year OS of patients with HCC were higher than those of age, gender, tumor grade, and tumor stage (**Figures 6K–O**). These results indicate that PANRS is a reliable predictive tool with better predictive performance than common clinical indicators. In particular, the individualized predictive performance suggests the potential clinical value of PANRS.

### 3.5 Validation of the prognostic effect of PANRS in the ICGC-LIRI cohort and comparison with previously reported signatures

**Figure 7:**
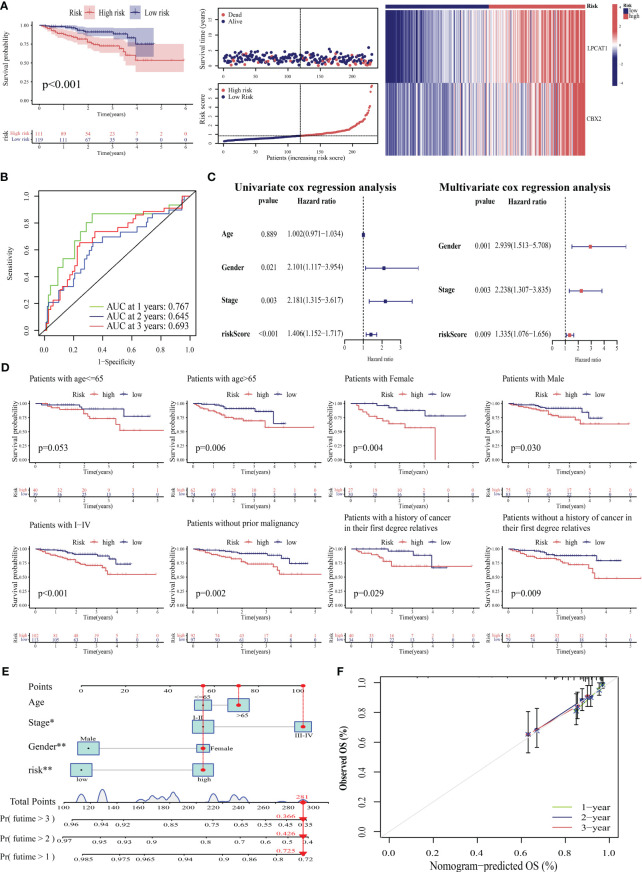
Verification of the prognostic predictive performance of PANoptosis-related signature (PANRS) in an external hepatocellular carcinoma (HCC) cohort. **(A)** Differential overall survival (OS) and risk curve between the high-risk and low-risk groups in the International Cancer Genome Consortium-Liver cancer-Riken-Japan (ICGC-LIRI-JP) cohort; **(B)** PANRS predicted the area under the curve (AUC) values for 1-year, 2-year, and 3-year OS in patients with HCC in the ICGC-LIRI-JP cohort; **(C)** Verification of the independent prognostic value of PANRS in the ICGC-LIRI-JP cohort; **(D)** Assessment of the clinical applicability of PANRS in the ICGC-LIRI-JP cohort; **(E, F)** The nomogram **(E)** and its prediction accuracy **(F)** in the ICGC-LIRI-JP cohort.

External validation was performed to clarify the generalizability of PANRS. The ICGC-LIRI cohort was divided into high-risk and low-risk groups based on the median risk score of TCGA-LIHC training cohort. As shown in **Figure 7A**, the high-risk group exhibited poor OS (*p* < 0.001) and upregulated expression of poor prognosis-related genes (*LPCAT1* and *CBX2*). The AUC values of PANRS for predicting the 1-year, 2-year, and 3-year OS of patients with HCC were approximately 0.7 (**Figure 7B**). Independent prognostic analysis, clinical applicability analysis, nomogram, and calibration charts confirmed that the risk scores can independently, accurately, and individually predict the survival of patients in the ICGC-LIRI cohort for a wide range of populations (all *p* < 0.05, **Figures 7C–F**). Clinical correlation analysis revealed that the expression of the unfavorable prognostic gene *LPCAT1* in patients with stage III–IV tumors was markedly higher than that in patients with stage I–II tumors (**Figure S2A**). Additionally, expression of *CBX2* was upregulated in patients with a positive family history of tumors in first-degree relatives (*p* = 0.027, **Figure S2B**).

This study also compared the prognostic performance of PANRS with that of previously reported signatures by determining the AUC values. The AUC values of PANRS for predicting the 1-year, 2-year, 3-year, and 5-year OS were higher than those of amino acid metabolism-related gene signature (45), basement membrane-related gene signature (46), and bile acid-related prognostic signature (47) (**Figures S2C–F**). This indicates that the predictive power of PANRS is higher than that of previously reported signatures.

### 3.6 PANRS can predict the prognosis of patients with kidney renal papillary cell carcinoma

Based on the prognostic power of the risk score for HCC determined in this study, the prognostic power of PANRS for 31 other tumors in TCGA database was examined. The median risk score of PANRS was used to divide TCGA-KIRP cohort into high-risk (n = 196) and low-risk (n = 82) groups. The OS of the high-risk group was significantly lower OS than that of the low-risk group (**Figure S2G**). The AUC values of PANRS for predicting the 1-year, 2-year, and 3-year OS in patients with KIRP were 0.729, 0.746, and 0.712, respectively (**Figure S2H**). The risk scores of patients with KIRP were combined with their clinical characteristics to obtain a nomogram (**Figure S2I**) for assessing the individual survival probability. The survival probability predicted using the nomogram was highly consistent with the actual survival probability, indicating that this nomogram can accurately predict the survival probability of KIRP patients with KIRP exhibiting different clinical characteristics (**Figure S2J**). Additionally, the C-index and AUC values of the nomogram were higher than those of age, sex, and tumor stage, except in the first year (**Figures S2K–O**). These results suggest that PANRS is a powerful prognostic tool whose application may not be limited to HCC.

### 3.7 PANRS can accurately predict the immune landscape and immunotherapy response in patients with HCC

**Figure 8:**
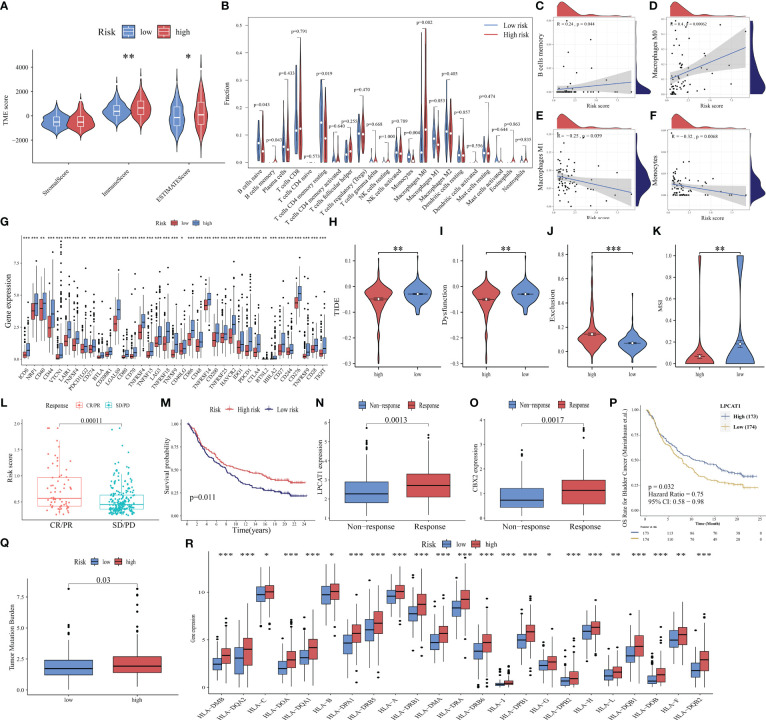
Ability of PANoptosis-related signature (PANRS) to predict immune landscape and immunotherapy response in hepatocellular carcinoma (HCC). **(A)** Differential tumor microenvironment (TME) between the high-risk and low-risk groups; **(B)** Comparative analysis of immune cell infiltration status between the high-risk and low-risk groups; **(C–F)** The correlation of risk scores with memory B cells **(C)**, M0 macrophages **(D)**, M1 macrophages **(E)**, and monocytes **(F)**; **(G)** Differential expression of immune checkpoint molecules between the high-risk and low-risk groups; **(H–K)** Comparative analysis of tumor immune dysfunction and exclusion (TIDE) **(H)**, dysfunction **(I)**, exclusion **(J)**, and microsatellite instability (MSI) scores **(K)** between the high-risk and low-risk groups; **(L)** Analysis of the correlation of risk scores with complete response (CR)/partial response (PR) and stable disease (SD)/progressive disease (PD) in the IMvigor210 cohort; **(M)** Comparison of overall survival (OS) between the high-risk and low-risk group in the IMvigor210 cohort; **(N, O)** The predictive value of the expression of the signature genes *LPCAT1* **(N)** and *CBX2* **(O)** for immunotherapy response; **(P)** Effect of *LPCAT1* expression levels on the OS of patients with bladder cancer; **(Q)** Distribution of tumor mutational burden (TMB) in different risk groups; **(R)** Differential expression of human leukocyte antigen (HLA) molecules in different risk groups. ^*^ *p* < 0.05, ^**^ *p* < 0.01, and ^***^ *p* < 0.001.

The tumor microenvironment (TME) is a multifaceted and dynamic ecosystem that significantly affects cancer progression, as well as the efficacy of both immunotherapy and drug treatments (48). The ESTIMATE algorithm was used to obtain the stromal, immune, and ESTIMATE scores. The immune and ESTIMATE scores of the high-risk group were markedly higher than those of the low-risk group (all *p* < 0.05, **Figure 8A**). This suggests an increased infiltration of immune cells in the TME of the high-risk group. Further analysis revealed that the high-risk group exhibited an increased abundance of memory B cells and M0 macrophages, which were both positively correlated with the risk score (**Figures 8B–D**). Conversely, the abundance of naïve B cells, resting memory CD4 T cells, and monocytes was downregulated in the high-risk group. M1 macrophages and monocytes were negatively correlated with the risk score (**Figures 8E, F**). These findings indicated that the high-risk group is highly susceptible to immunosuppressive microenvironments.

ICI, a promising therapeutic for cancer, exerts growth-inhibitory effects against tumor cells by improving the immune function of patients. Only a small proportion of patients benefit from the approved ICIs. The identification of new immune checkpoints can aid in improving the immunotherapy response of patients (49). In this study, the differential expression of 40 immune checkpoint genes (50, 51) in different risk groups was analyzed. The expression levels of 37 immune checkpoints, except for adenosine A2a receptor (*ADORA2A*), inducible T cell costimulator ligand (*ICOSLG*), and tumor necrosis factor superfamily 14(*TNFSF14*), in the high-risk group were higher than those in the low-risk group (**Figure 8G**). Further validation with the TIDE algorithm demonstrated that the high-risk group exhibited decreased TIDE, dysfunction, and MSI scores but increased exclusion scores (*p* < 0.05; **Figures 8H–K**). This suggests a reduced likelihood of tumor immune escape and a potentially favorable response to immunotherapy in the high-risk group.

These findings were further supported by the analysis of the IMvigor210 cohort. The risk scores of the immunotherapy response group (complete remission/partial remission) were markedly higher than those of the immunotherapy non-response group (stable disease/progressive disease) (**Figure 8L**). Additionally, the OS of the high-risk group was higher than that of the low-risk group after immunotherapy (*p* = 0.011, **Figure 8M**), indicating that patients with high risk scores responded well to immunotherapy. The expression levels of the model genes *LPCAT1* and *CBX2* in the immunotherapy response group were higher than those in the non-responsive group (all *p* < 0.01, **Figures 8N, O**). The upregulated expression of *LPCAT1* could also predict a positive response to immunotherapy in patients with bladder cancer (*p* = 0.032, **Figure 8P**).

Additionally, TMB (52) and MHC molecules (24) can predict immunotherapy response. Neoantigens, which are produced by tumors with high TMB, are often associated with improved immunotherapeutic outcomes. However, the downregulation of MHC molecules may suppress the recognition of neoantigens by T cells and consequently decrease the efficacy of immunotherapy (53). This study investigated TMB and MHC expression in different risk groups and demonstrated that increased TMB was positively correlated with the high-risk group. The expression levels of 95.83% (23/24) of the examined HLA genes were significantly upregulated in the high-risk group (*p* < 0.05, **Figures 8Q, R**). These findings suggest that PANRS represents a novel and effective signature for predicting immunotherapy response.

### 3.8 Chemotherapy drug sensitivity of HCC can be predicted using PANRS

**Figure 9:**
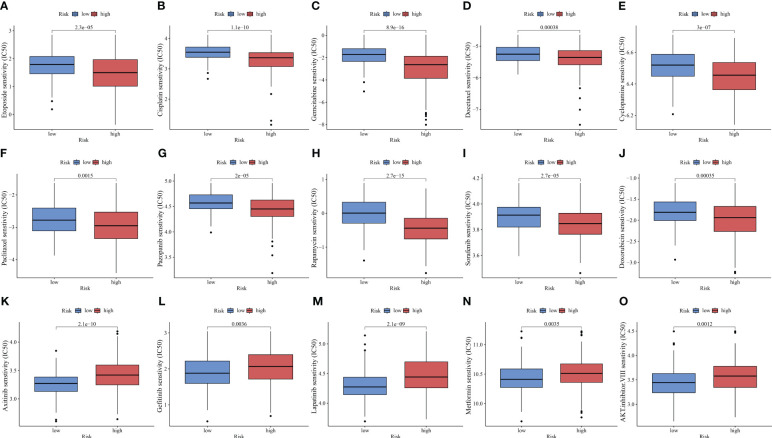
PANoptosis-related signature (PANRS) predicts the sensitivity of hepatocellular carcinoma to common chemotherapy drugs. **(A–J)** Chemotherapy drugs to which the high-risk group is sensitive relative to the low-risk group; **(K–O)** Chemotherapy drugs to which the low-risk group is sensitive relative to the high-risk group.

ICIs have improved the clinical outcomes of patients with HCC, increasing the survival rate of patients who responded to the therapy. However, multi-drug resistance mechanisms are the major limiting factors for the efficacy of ICIs (54). Combination therapy, including chemotherapy, is still the main treatment modality. This study compared the IC50 values to predict the sensitivity of HCC populations with different risks to chemotherapeutic drugs. The high-risk group exhibited increased sensitivity to several drugs, including etoposide, cisplatin, gemcitabine, docetaxel, cyclopamine, paclitaxel, pazopanib, rapamycin, sorafenib, and doxorubicin (*p* < 0.01, **Figures 9A–J**). Meanwhile, the low-risk group was sensitive to axitinib, gefitinib, lapatinib, metformin, and AKT inhibitor VIII (*p* < 0.01, **Figures 9K–O**).

### 3.9 Identification of the carcinogenic effect of *LPCAT1* on HCC

**Figure 10:**
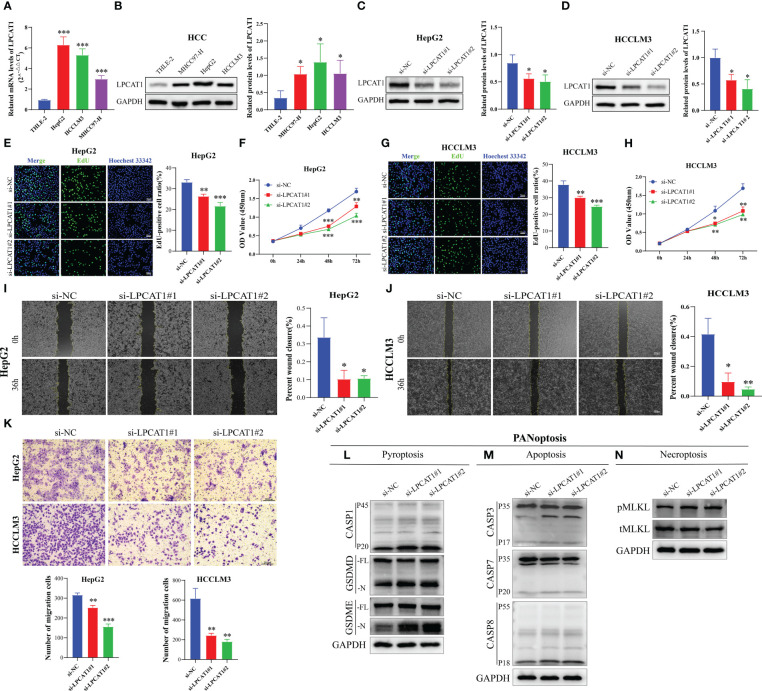
*LPCAT1* knockdown inhibits hepatocellular carcinoma (HCC) cell proliferation and migration and promotes PANoptosis. **(A)** Quantitative real-time polymerase chain reaction analysis of *LPCAT1* mRNA expression in healthy liver cell lines (THLE-2) and HCC cell lines (HCCLM3, MHCC97-H, and HepG2); **(B)** Western blotting analysis of *LPCAT1* protein expression levels in healthy liver cell lines (THLE-2) and HCC cell lines (HCCLM3, MHCC97-H, and HepG2); GAPDH was used as an internal control; **(C, D)** The knockdown efficiency of different siRNA-*LPCAT1* constructs in HepG2 and HCCLM3 cells was evaluated using western blotting; **(E–H)** Effects of transfection with siRNA–*LPCAT1*#1, siRNA–*LPCAT1*#2 or negative control (si-NC) on cell proliferation were assessed using ethynyl-deoxyuridine (EdU) and cell counting kit (CCK)-8 assays; **(E, G)** Representative images of the changes in the number of proliferating HepG2 and HCCLM3 cells in different groups after transfection and quantitative analysis of EdU-positive rate (original magnification, ×200; scale bar, 50 μm); **(F, H)** The line graphs show the changes in the viability of HepG2 and HCCLM3 cells in different groups at 0, 24, 48, and 72 h post-transfection; **(I, J)** Cell migration was examined using the wound-healing assay. Representative images and quantitative analysis of wound closure area in different groups at 0 and 36 h post-scratching are presented (original magnification, ×40; scale bar, 200 μm); **(K)** Transwell assay was used to evaluate the migration ability of transfected HCC cells. The upper panel shows representative images (original magnification, ×200; scale bar, 200 μm), while the lower panel (histogram) shows the number of migrated cells in different groups; **(L-N)** Immunoblotting analysis of **(L)** pro-CASP1 (P45) and activated (P20) CASP1, pro-GSDMD (-FL), and activated GSDMD (-N); and pro-GSDME (-FL) and activated (-N) GSDME; **(M)** pro-CASP3 (P35) and cleaved (P17) CASP3, pro-CASP7 (P35) and cleaved (P20) CASP7, and pro-CASP8 (P55) and cleaved (P18) CASP8; and **(N)** phosphorylated MLKL (pMLKL), and total MLKL (tMLKL) in HCCLM3 cells transfected with si-NC, si-*LPCAT1*#1, and si-*LPCAT1*#2. GAPDH was used as the internal control. Data are representative of at least three independent experiments.^*^ *p* < 0.05, ^**^ *p* < 0.01, and ^***^ *p* < 0.001.

In our PANRS, *LPCAT1* has a higher risk coefficient. Consider that the sequencing data we previously analyzed were all at the transcriptome level. We therefore assessed the protein expression level of *LPCAT1* using the Clinical Proteomic Tumor Analysis Consortium (CPTAC) database. The notable overexpression of *LPCAT1* protein in HCC, as shown in **Figure S1**, indicates strong consistency between *LPCAT1* mRNA and protein expression. However, whether the effect of *LPCAT1* on hepatoma cells is consistent with our results still needs to be verified by further *in vitro* experiments. By qRT-PCR and western blotting, we found that compared with normal hepatocytes THLE-2, mRNA and protein of *LPCAT1* were substantially overexpressed in HCC cells (MHCC97-H, HepG2, and HCCLM3) (all *p* < 0.05, **Figures 10A, B**). Furthermore, RNA interference was performed on HepG2 and HCCLM3 with high expression levels. As shown in **Figures 10C, D**, we successfully inhibited the expression of *LPCAT1* protein in HCC cells. By EdU staining and CCK-8 assay, the results indicated that interference with *LPCAT1* expression significantly inhibited the proliferative activity of HCC cells (all *p* < 0.05, **Figures 10E–H**). Further analysis using wound-healing and transwell assays revealed a significant decrease in HCC cell migration following interference with *LPCAT1* expression (all *p* < 0.05, **Figures 10I–K**). These findings indicate that *LPCAT1* contributes to the progression of HCC.

To investigate the effect of *LPCAT1* knockdown on pyroptosis, we performed pyroptosis induction in HCCLM3 cells using LPS and ATP and examined the expression level of cleaved GSDMD. *LPCAT1* knockdown in HCCLM3 cells promoted the cleavage of GSDMD, generating bioactive GSDMD-N fragments, which can form membrane pores to initiate cellular pyroptosis (**Figure 10L**). In the canonical inflammasome-mediated pyroptosis process, GSDMD is cleaved by activated CASP1, leading to the release of GSDMD-N fragments (55, 56). Consistent with the generation of GSDMD-N, *LPCAT1* knockdown upregulated the levels of cleaved CASP1 (p20) (**Figure 10L**). GSDME, a member of the Gasdermin family, is reported to induce pyroptosis under specific conditions (57). *LPCAT1* knockdown promoted the cleavage of GSDME (**Figure 10L**). In addition to pyroptosis, *LPCAT1* knockdown promoted apoptosis in HCCLM3 cells as evidenced by the minor cleavage of CASP3 (p17), CASP7 (p20), and CASP8 (p18) (**Figure 10M**). Finally, the effect of *LPCAT1* knockdown on necroptosis was examined. *LPCAT1* knockdown upregulated MLKL phosphorylation in HCCLM3 cells, indicating the induction of necroptosis (**Figure 10N**). These findings indicate that *LPCAT1* knockdown promotes PANoptosis in cells.

## 4 Discussion

HCC, a fatal malignancy with high incidence rates, is diagnosed at an advanced stage. Thus, most patients with HCC are not eligible for curative treatments, such as liver transplantation and surgical resection. Sorafenib and lenvatinib are the only first-line systemic therapies for HCC. Second-line treatments are generally limited and ineffective (58, 59). Therefore, effective strategies must be developed to mitigate HCC-related mortality rates. Recently, the induction and regulation of inflammatory cell death have emerged as a novel anticancer therapeutic strategies as it elicits an immune response and stimulates strong anticancer effect (60). This enabled the investigators to analyze the correlation of HCC with pyroptosis, apoptosis, and necroptosis and identify potential biomarkers (61–66). The three PCD pathways can complement each other during PANoptosis, responding to specific stimuli in the surrounding TME. Therefore, these pathways function cooperatively to achieve immunogenic PCD (19). For example, in patients undergoing immunotherapy, PANoptosis kills cancer cells by activating alternative PCD pathways, such as pyroptosis or necroptosis if cancer cells inhibit apoptosis. Treatment with the combination of PANoptosis inducers and ICIs exerted potent growth-inhibitory effects even against ICI-resistant tumors (67). As HCC is associated with drug resistance, this study developed a prognostic signature based on the molecular subtype of PANoptosis for patients with HCC. This study demonstrated that this signature can predict the prognosis and immunotherapy response of patients with HCC, providing a new direction for precise individualized therapy.

This study identified three PANoptosisClusters for HCC. Compared with those in PANoptosisClusters B and C, the OS was lower and the levels of infiltrating immune cells were higher in PANoptosisCluster A. Moreover, PANoptosisCluster A was enriched in Fc γ receptor (Fc-γR)-mediated phagocytosis when compared with PANoptosisClusters B and C. Monoclonal antibodies (Mabs) are one of the most well-known targeted therapies for various types of malignant tumors. IgG Fc can help clinically approved Mabs achieve optimal efficacy through interaction with Fc-γR. The phagocytosis of antibody-bound tumor cells by macrophages *via* antibody-dependent cellular phagocytosis (ADCP) is one of the major mechanisms underlying Fc-γR-mediated tumor immune response (68). This indicates that PANoptosisCluster A with high levels of infiltrating immune cells is more likely to elicit tumor immune response through ADCP than other subtypes, contributing to enhanced immunotherapeutic effects. However, the upregulation of FC-γRIIB (single inhibitory FC-γR) under hypoxic conditions may confer resistance to this therapy (69).

To facilitate prognosis prediction in patients with HCC, DEGs between three PANoptosisClusters were screened. These DEGs were associated with prognosis. Subsequently, a PANRS was constructed for the training cohort. The predictive accuracy, independent predictability, and population suitability of PANRS were validated using TCGA-LIHC and ICGC-LIRI-JP cohorts. To further determine the clinical application value of PANRS, a nomogram was generated for patients with HCC to predict their specific survival probability by combining risk scores with age, gender, and tumor stage. The calibration curves and C-index maps confirmed the high accuracy of this comprehensive risk score for predicting the survival probabilities of patients with HCC in both TCGA-LIHC and ICGC-LIRI-JP cohorts. Thus, PANRS is a reliable and effective tool for predicting HCC prognosis.

Next, the efficacy of PANRS in predicting the immune landscape and immunotherapy response of HCC was examined. Patients in the high-risk group exhibited increased levels of infiltrating memory B cells and M0 macrophages, which promote HCC progression and immunosuppression (70). For example, M0 macrophages can be polarized into immunosuppressive M2 macrophages upon stimulation with tumor-derived alpha fetoprotein and inhibit M1 macrophages from phagocytosing HCC cells (71). Immune checkpoint molecules prevent aberrant activation of immune responses and maintain homeostasis. However, tumor cells use this characteristic of immune checkpoint molecules to escape the immune response. Therefore, ICIs, which mitigate immune evasion, are the fourth most frequent treatment modality for cancer after surgical interventions, chemotherapy, and radiotherapy (72). This study suggested that the high-risk group with increased expression of immune checkpoint genes may have a favorable response to ICI therapy. To verify this, the TIDE score, IMvigor210 immunotherapy cohort data, TMB, and HLA molecules were analyzed. The high-risk group exhibited upregulated levels of TMB and HLA molecules, increased incidence of complete response/partial response, and decreased TIDE score. A high TMB is strongly correlated with improved clinical outcomes in patients with HCC undergoing immunotherapy (73). The expression levels of MHC type I molecules (HLA-A, HLA-B, and HLA-C) determine the effectiveness of immunotherapy (74). A high TIDE score suggests an increased probability of tumor immune evasion (75). The predictive power of TIDE is higher than that of TMB and *CD274* in anti-PDCD1 or anti-CTLA4 therapy (75, 76). These findings suggest that patients in the high-risk group are favorably responsive to immunotherapy.

PANRS developed in this study comprised *LPCAT1* and *CBX2*. The findings of bioinformatics analysis of *LPCAT1*, which contributed the most to PANRS, were verified using *in vitro* experiments. *LPCAT1* expression in three HCC cell lines was significantly upregulated when compared with that in healthy liver cells. Additionally, *LPCAT1* knockdown significantly suppressed the proliferation and migration of HCC cells, indicating that *LPCAT1* exerts oncogenic effects in HCC and adversely affects patient prognosis. These observations are consistent with those of previous studies (77–79). The correlation between *LPCAT1* and PANoptosis has not been completely elucidated. However, *LPCAT1* is reported to be involved in pyroptosis and apoptosis in various cancers, including HCC, cervical cancer, endometrial cancer, and squamous cell carcinoma of the skin (80–83). *CBX2* expression is associated with cancer cell apoptosis. For example, *CBX2* suppresses apoptosis in high-grade serous ovarian cancer (HGSOC) cells (84). Consistently, the downregulation of *CBX2* significantly upregulated apoptosis in HCC, HGSOC, acute myeloid leukemia, and colorectal cancer cells (85–88). Furthermore, the research team of Ding reported that *CBX2* is involved in the formation of pyroptosis-related signature (80).

This study has some limitations. The ability of PANRS to predict HCC prognosis and ICI treatment response was determined in this study. However, the samples included in this study were retrospectively analyzed. Thus, large-scale clinical trials must be performed and prospective samples must be collected to further confirm that PANRS is an excellent and practical clinical prognostic tool. Additionally, the risk score in this study was dependent on the expression level of the signature gene but did not consider the impact of other factors, including gene mutations, on the prognosis of patients with HCC.

## 5 Conclusions

This study identified three molecular subtypes of PANoptosis in patients with HCC. The PANRS, which was constructed based on these subtypes, was demonstrated to be a reliable and independent tool for accurately predicting the prognosis and immunotherapy response of patients with HCC. Thus, the PANRS can contribute to the risk stratification of HCC and facilitate individualized immunotherapy.

## Data availability statement

The original contributions presented in the study are included in the article/**Supplementary Materials**, further inquiries can be directed to the corresponding author/s.

## Author contributions

JZ and QH wrote the manuscript, JZ, XP, JZ, CL, and ZL designed and completed the experiment. QH and DL collected, collated, and analyzed the data. JZ and QH drew the drawings. HY, RY, and XC conceived the study, made major revisions to the manuscript, and provided funding. All authors read and approved the final manuscript.
