# Supplementary material for: Identification of molecular subtypes based on PANoptosis-related genes and construction of a signature for predicting the prognosis and response to immunotherapy response in hepatocellular carcinoma
Source: Front Immunol. 2023 Aug 18;14:1218661. doi: 10.3389/fimmu.2023.1218661 (PMC10471990; doi:10.3389/fimmu.2023.1218661)
Supplement: Supplementary file 1 [file DataSheet_1.docx]

Supplementary Material

Identification of Molecular Subtypes Based on PANoptosis-related Genes and Construction of a Signature for Better Prediction of Prognosis and Response to Immunotherapy in Hepatocellular Carcinoma

Jinfeng Zhu^1†^, Qian Huang^2†^, Xingyu Peng^1^, Chen Luo^3^, Zitao Liu^1^, Dongdong Liu^4^, Huazhao Yuan^5^, Rongfa Yuan^1*^, Xuexin Cheng^6,7,8*^

**^†^**Jinfeng Zhu and Qian Huang contributed equally to this work.

*** Correspondence:**

Xuexin Cheng, Email: [cxxncu@163.com](mailto:cxxncu@163.com);

Rongfa Yuan, Email: [yuanrf7788@163.com](mailto:yuanrf7788@163.com).

**Supplementary Figures and Tables**

## Supplementary Figure


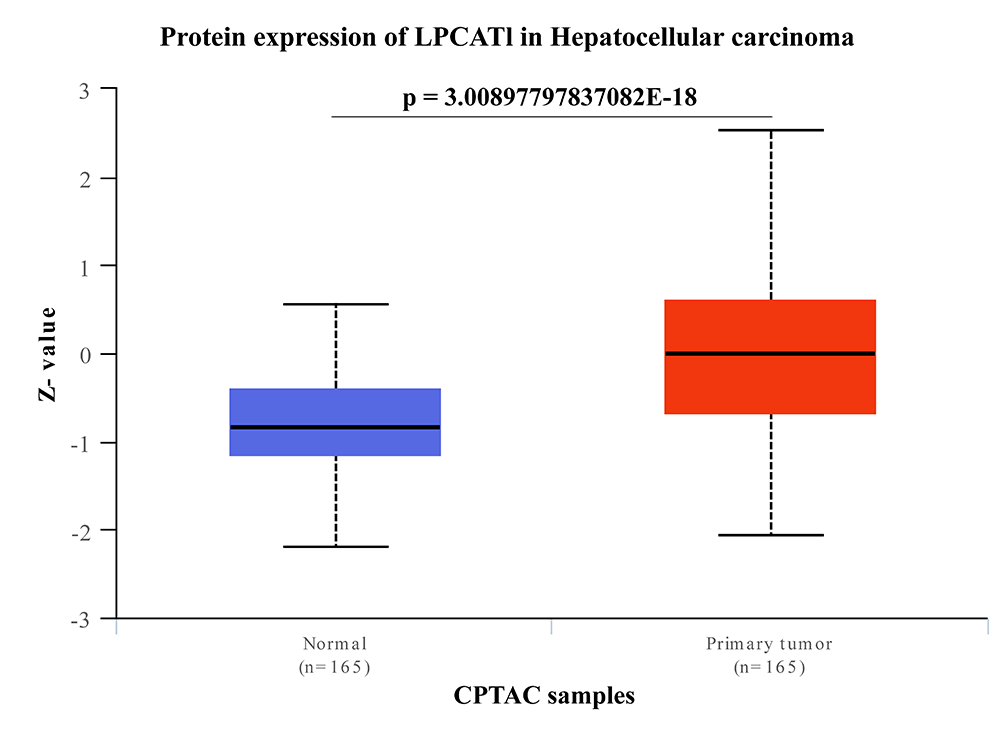


**Figure S1. Differential expression of *LPCAT1* in hepatocellular carcinoma (HCC) and healthy liver tissues.**

Differential expression of *LPCAT1* protein between primary HCC and healthy tissues was examined using the Clinical Proteomic Tumor Analysis Consortium dataset. Z-scores represent the standard deviation of the sample median for a given cancer type.


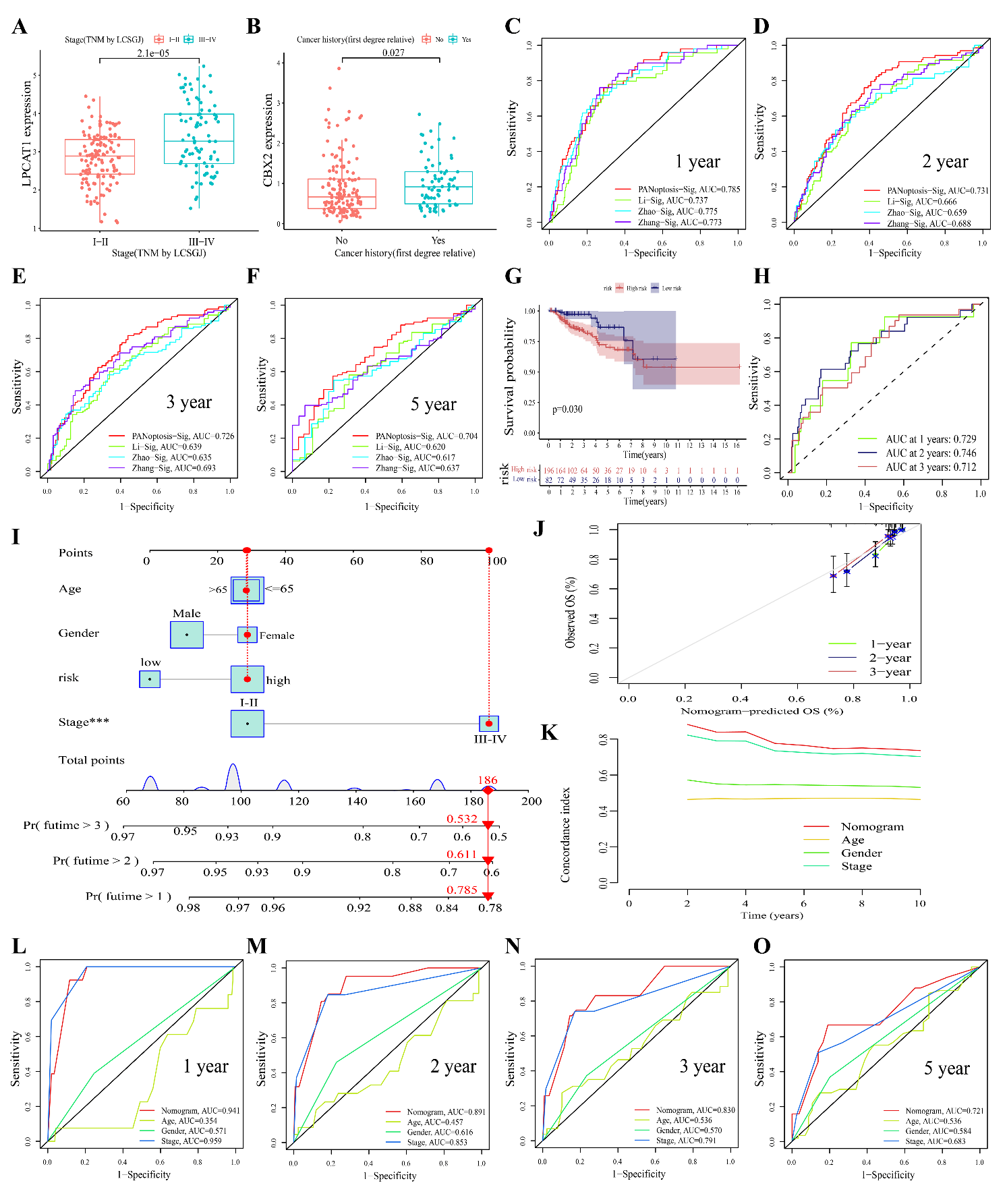


**Figure S2. Verification of the prognostic predictive performance of PANoptosis-related signature (PANRS) in an external hepatocellular carcinoma (HCC) cohort and a kidney renal papillary cell carcinoma (KIRP) cohort, and comparison with other HCC signatures.**

**(A)** The expression of *LPCAT1* according to tumor stages (LCSGJ); **(B)** The correlation between the expression of *CBX2* and the tumor history of first-degree relatives; **(C-F)** Comparison of the performance of PANRS with that of previously reported signatures for predicting 1-year **(C)**, 2-year **(D)**, 3-year **(E)**, and 5-year **(F)** OS in patients with HCC; **(G)** Differential OS between the high-risk and low-risk groups in The Cancer Genome Atlas (TCGA)-KIRP cohort; **(H)** PANRS predicted the AUC values for 1-year, 2-year, and 3-year OS in patients with HCC; **(I)** A nomogram was constructed by combining the risk scores with age, sex, and tumor stage in TCGA-KIRP cohort; **(J, K)** Calibration curve **(J)** and C-index **(K)** confirmed the high accuracy of the nomogram; **(L-O)** The receiver operating characteristic (ROC) curve was used to compare the ability of the nomogram with other common clinical indicators in predicting the 1-year **(L)**, 2-year **(M)**, 3-year **(N)** and 5-year **(O)** OS of patients with HCC.

## Supplementary Tables

**Table S1. PANoptosis gene list**

| **Gene** | Type |
| --- | --- |
| **AIFM1** | Apoptosis |
| **AKT3** | Apoptosis |
| **APAF1** | Apoptosis |
| **APPL1** | Apoptosis |
| **BMF** | Apoptosis |
| **BNIP3L** | Apoptosis |
| **BOK** | Apoptosis |
| **CASP10** | Apoptosis |
| **CASP2** | Apoptosis |
| **CASP6** | Apoptosis |
| **CASP7** | Apoptosis |
| **CASP8** | Apoptosis |
| **CD14** | Apoptosis |
| **CHUK** | Apoptosis |
| **CRADD** | Apoptosis |
| **DFFA** | Apoptosis |
| **DFFB** | Apoptosis |
| **E2F1** | Apoptosis |
| **HMGB2** | Apoptosis |
| **IGF1** | Apoptosis |
| **LY96** | Apoptosis |
| **PPP3R1** | Apoptosis |
| **TFDP1** | Apoptosis |
| **TICAM1** | Apoptosis |
| **TNFRSF1A** | Apoptosis |
| **TNFSF10** | Apoptosis |
| **TP73** | Apoptosis |
| **TRAF2** | Apoptosis |
| **UACA** | Apoptosis |
| **UNC5B** | Apoptosis |
| **YWHAE** | Apoptosis |
| **YWHAG** | Apoptosis |
| **FADD** | Necroptosis |
| **FAS** | Necroptosis |
| **FASLG** | Necroptosis |
| **MLKL** | Necroptosis |
| **RIPK1** | Necroptosis |
| **RIPK3** | Necroptosis |
| **TLR3** | Necroptosis |
| **TNF** | Necroptosis |
| **BAK1** | Pyroptosis |
| **BAX** | Pyroptosis |
| **CASP1** | Pyroptosis |
| **CASP3** | Pyroptosis |
| **CASP4** | Pyroptosis |
| **CASP5** | Pyroptosis |
| **CHMP2A** | Pyroptosis |
| **CHMP2B** | Pyroptosis |
| **CHMP3** | Pyroptosis |
| **CHMP4A** | Pyroptosis |
| **CHMP4B** | Pyroptosis |
| **CHMP4C** | Pyroptosis |
| **CHMP6** | Pyroptosis |
| **CHMP7** | Pyroptosis |
| **CYCS** | Pyroptosis |
| **ELANE** | Pyroptosis |
| **GSDMD** | Pyroptosis |
| **GSDME** | Pyroptosis |
| **GZMB** | Pyroptosis |
| **HMGB1** | Pyroptosis |
| **IL18** | Pyroptosis |
| **IL1A** | Pyroptosis |
| **IL1B** | Pyroptosis |
| **IRF1** | Pyroptosis |
| **IRF2** | Pyroptosis |

**Table S2. Primers and siRNA target sequences.**

| **Name** | **Sequences** |
| --- | --- |
| **Primers for real-time PCR:** |  |
| LPCAT1 sense | 5'-ACATCCCGATCTGGGGAACT-3' |
| LPCAT1 antisense | 5'-GGCCACTTTCCGTTGGACT-3' |
| GAPDH sense: | 5'-AGAAGGCTGGGGCTCATTTG-3' |
| GAPDH antisense: | 5'-AGGGGCCATCCACAGTCTTC-3' |
| The target sites of siRNA: |  |
| si-LPCAT#1 sense | 5'-CCUGCCUAAUUACCUUCAATT-3′ |
| si-LPCAT#1 antisense | 5'-UUGAAGGUAAUUAGGCAGGTT-3′ |
| si-LPCAT#2 sense | 5'-GGAGGAAGGUUGUGGACUUTT-3′ |
| si-LPCAT#2 antisense | 5'-AAGUCCACAACCUUCCUCCTT-3′ |
| si-NC sense | 5'-UUCUCCGAACGUGUCACGUTT-3′ |
| si-NC antisense | 5'-ACGUGACACGUUCGGAGAATT-3′ |

**Table S3. Differentially expressed genes associated with prognosis.**

| **id** | **HR** | **HR.95L** | **HR.95H** | **pvalue** |
| --- | --- | --- | --- | --- |
| **CBX2** | 1.622494192 | 1.397629734 | 1.883537063 | 2.04E-10 |
| **NEIL3** | 1.74931323 | 1.469555009 | 2.08232884 | 3.18E-10 |
| **G6PD** | 1.410106381 | 1.266186196 | 1.570385156 | 3.93E-10 |
| **KPNA2** | 1.730980086 | 1.451495486 | 2.064279282 | 1.01E-09 |
| **TRIP13** | 1.547555152 | 1.344771126 | 1.780917885 | 1.10E-09 |
| **CEP55** | 1.542707941 | 1.339178457 | 1.77717001 | 1.90E-09 |
| **PSRC1** | 1.733908556 | 1.448394866 | 2.075703906 | 2.03E-09 |
| **CENPA** | 1.5524934 | 1.344304455 | 1.792924026 | 2.13E-09 |
| **SGO2** | 1.832851531 | 1.502458485 | 2.235898541 | 2.31E-09 |
| **KIF2C** | 1.508197571 | 1.317632017 | 1.726324105 | 2.49E-09 |
| **MCM10** | 1.691543511 | 1.422328949 | 2.011714275 | 2.79E-09 |
| **PRR11** | 1.627067596 | 1.38558718 | 1.910633267 | 2.87E-09 |
| **CDCA8** | 1.534575124 | 1.331231026 | 1.768979813 | 3.53E-09 |
| **PFKFB4** | 1.642909568 | 1.393215299 | 1.937354441 | 3.58E-09 |
| **LPCAT1** | 1.489010672 | 1.304297337 | 1.699882933 | 3.83E-09 |
| **TAF3** | 2.239486086 | 1.712575373 | 2.928512233 | 3.84E-09 |
| **SLC1A5** | 1.363881772 | 1.229130092 | 1.513406515 | 5.01E-09 |
| **TTK** | 1.603842274 | 1.367511221 | 1.881015673 | 6.31E-09 |
| **GAS2L3** | 1.690783704 | 1.414654853 | 2.020810609 | 7.79E-09 |
| **CDC20** | 1.389904225 | 1.241557921 | 1.555975538 | 1.08E-08 |
| **KIF18A** | 1.685729995 | 1.409045954 | 2.016744455 | 1.14E-08 |
| **KIAA1841** | 2.254591062 | 1.702738363 | 2.985297664 | 1.38E-08 |
| **KIF20A** | 1.51273532 | 1.309502123 | 1.747510072 | 1.87E-08 |
| **CIP2A** | 1.664276475 | 1.393255792 | 1.988016989 | 1.94E-08 |
| **RBM17** | 2.126359755 | 1.631532591 | 2.771262942 | 2.38E-08 |
| **ERCC6L** | 1.888493344 | 1.51049958 | 2.361077856 | 2.41E-08 |
| **GTPBP4** | 2.016664085 | 1.57567098 | 2.581080746 | 2.53E-08 |
| **CLSPN** | 1.790437319 | 1.457955666 | 2.198740242 | 2.74E-08 |
| **NDC80** | 1.570340368 | 1.339233145 | 1.841329033 | 2.76E-08 |
| **SAPCD2** | 1.622989284 | 1.367864894 | 1.925697652 | 2.86E-08 |
| **DLGAP5** | 1.506837723 | 1.303544804 | 1.741834969 | 2.94E-08 |
| **DBF4** | 1.95771174 | 1.54280899 | 2.484192976 | 3.24E-08 |
| **PLK1** | 1.495637932 | 1.295568884 | 1.726602769 | 3.92E-08 |
| **NDC1** | 1.928921088 | 1.524912808 | 2.43996676 | 4.29E-08 |
| **SPINDOC** | 1.65034175 | 1.379529792 | 1.97431611 | 4.30E-08 |
| **TPX2** | 1.449057026 | 1.268623065 | 1.655153782 | 4.58E-08 |
| **CENPE** | 1.728528705 | 1.420380344 | 2.103529168 | 4.68E-08 |
| **ANLN** | 1.46010954 | 1.273742258 | 1.673745105 | 5.55E-08 |
| **HJURP** | 1.507139547 | 1.298864451 | 1.748811904 | 6.44E-08 |
| **CDCA2** | 1.604142274 | 1.350768384 | 1.905043429 | 7.13E-08 |
| **SFPQ** | 2.070104154 | 1.587495215 | 2.699429372 | 7.77E-08 |
| **ZNF239** | 1.563796819 | 1.327082769 | 1.842733963 | 9.34E-08 |
| **EZH2** | 1.63757237 | 1.366145701 | 1.962926255 | 9.59E-08 |
| **MACIR** | 1.471451833 | 1.276574913 | 1.696077899 | 9.89E-08 |
| **HDAC2** | 1.923028474 | 1.509055078 | 2.450565631 | 1.24E-07 |
| **RAN** | 1.924534438 | 1.509680753 | 2.453388105 | 1.26E-07 |
| **IMPDH1** | 1.427866184 | 1.250712656 | 1.630112103 | 1.36E-07 |
| **UBE2S** | 1.518480176 | 1.299523978 | 1.774328204 | 1.46E-07 |
| **DEPDC1** | 1.542614095 | 1.312436869 | 1.813160162 | 1.46E-07 |
| **CENPO** | 1.780840496 | 1.435641056 | 2.209043034 | 1.53E-07 |
| **OLA1** | 2.089316552 | 1.585817641 | 2.752676941 | 1.63E-07 |
| **TMEM237** | 1.819306047 | 1.453900563 | 2.276548049 | 1.68E-07 |
| **CHEK1** | 1.586274681 | 1.3337856 | 1.886560602 | 1.83E-07 |
| **SKA1** | 1.438621763 | 1.25478702 | 1.649389534 | 1.85E-07 |
| **MTMR2** | 1.900259392 | 1.491958466 | 2.420299116 | 1.98E-07 |
| **CENPI** | 1.677895707 | 1.379075211 | 2.041465167 | 2.32E-07 |
| **CCNB1** | 1.446553721 | 1.257399257 | 1.664163277 | 2.43E-07 |
| **TPD52L2** | 1.95674538 | 1.516367154 | 2.525016762 | 2.47E-07 |
| **NCAPG** | 1.487399229 | 1.27918319 | 1.729507146 | 2.47E-07 |
| **GTSE1** | 1.4830133 | 1.27647283 | 1.722973178 | 2.60E-07 |
| **ORC6** | 1.595021394 | 1.33529488 | 1.905266983 | 2.62E-07 |
| **ELOVL1** | 1.937911056 | 1.505915951 | 2.493830587 | 2.73E-07 |
| **SPC25** | 1.533389308 | 1.301959872 | 1.805956404 | 3.04E-07 |
| **FBXO5** | 1.678618984 | 1.376598693 | 2.046901329 | 3.09E-07 |
| **SAP30** | 1.740624996 | 1.407654615 | 2.15235708 | 3.12E-07 |
| **CCT5** | 1.778696203 | 1.424911002 | 2.220321255 | 3.59E-07 |
| **CCT6A** | 2.032037854 | 1.546284797 | 2.670386366 | 3.64E-07 |
| **NOP56** | 1.714975441 | 1.393072521 | 2.111261773 | 3.67E-07 |
| **AMD1** | 1.865285834 | 1.465563126 | 2.374030282 | 4.06E-07 |
| **SLC16A3** | 1.364353855 | 1.209818319 | 1.53862891 | 4.07E-07 |
| **TRNP1** | 1.267010258 | 1.155921421 | 1.388775192 | 4.31E-07 |
| **BUB1** | 1.508663434 | 1.286034082 | 1.769832845 | 4.46E-07 |
| **CKS2** | 1.545318045 | 1.304980279 | 1.829918734 | 4.50E-07 |
| **HDAC1** | 1.940603838 | 1.499780891 | 2.510995625 | 4.59E-07 |
| **GINS1** | 1.454448045 | 1.257091527 | 1.68278846 | 4.77E-07 |
| **GNPDA1** | 1.703048914 | 1.383194892 | 2.096866913 | 5.27E-07 |
| **ORC1** | 1.50199027 | 1.281135986 | 1.760917496 | 5.36E-07 |
| **CDCA3** | 1.517671613 | 1.289013532 | 1.786891345 | 5.53E-07 |
| **NUF2** | 1.418984346 | 1.237142211 | 1.627554663 | 5.69E-07 |
| **MYBL2** | 1.286422648 | 1.165333516 | 1.420094082 | 5.93E-07 |
| **WDHD1** | 1.661347758 | 1.359988502 | 2.029485078 | 6.67E-07 |
| **KIF15** | 1.524969106 | 1.290765029 | 1.801668562 | 7.04E-07 |
| **KIF23** | 1.479963202 | 1.267476925 | 1.72807176 | 7.14E-07 |
| **NCBP2** | 2.003528557 | 1.521608687 | 2.638080811 | 7.41E-07 |
| **PBK** | 1.40626736 | 1.228639933 | 1.60957481 | 7.47E-07 |
| **MELK** | 1.43070867 | 1.241365247 | 1.648932338 | 7.61E-07 |
| **CSTF2** | 1.692562412 | 1.373503863 | 2.085736775 | 7.90E-07 |
| **BIRC5** | 1.353770798 | 1.200346067 | 1.526805829 | 7.99E-07 |
| **STMN1** | 1.442464438 | 1.247042115 | 1.668511137 | 8.13E-07 |
| **CCT2** | 1.957742672 | 1.497959713 | 2.558651169 | 8.71E-07 |
| **KIF18B** | 1.418974589 | 1.234170867 | 1.631450666 | 8.86E-07 |
| **PRPF38A** | 1.982974152 | 1.508873227 | 2.606041658 | 9.07E-07 |
| **LMNB2** | 1.546752673 | 1.298868102 | 1.841945172 | 9.87E-07 |
| **EXO1** | 1.520838412 | 1.285635234 | 1.799071319 | 1.00E-06 |
| **SPATS2** | 1.708148398 | 1.377967721 | 2.117445063 | 1.03E-06 |
| **KIF4A** | 1.381308189 | 1.212933956 | 1.573055402 | 1.11E-06 |
| **E2F2** | 1.674462808 | 1.360422723 | 2.060995928 | 1.15E-06 |
| **PFN2** | 1.305292047 | 1.171996407 | 1.453747912 | 1.25E-06 |
| **RIT1** | 1.742470783 | 1.391954969 | 2.181251905 | 1.26E-06 |
| **ATP13A2** | 1.606906843 | 1.326078883 | 1.947206638 | 1.30E-06 |
| **MCM6** | 1.49822998 | 1.271902203 | 1.764831501 | 1.31E-06 |
| **IQGAP3** | 1.455633474 | 1.249862239 | 1.695281883 | 1.38E-06 |
| **PTTG1** | 1.37856904 | 1.210045045 | 1.570563514 | 1.39E-06 |
| **REEP4** | 1.578076242 | 1.311156422 | 1.899334501 | 1.40E-06 |
| **ZWINT** | 1.418902917 | 1.23073119 | 1.63584502 | 1.44E-06 |
| **GIT1** | 1.777347911 | 1.406221335 | 2.246421325 | 1.49E-06 |
| **RAD54L** | 1.58747948 | 1.314987403 | 1.916437446 | 1.51E-06 |
| **CAD** | 1.658962458 | 1.349393417 | 2.03955081 | 1.56E-06 |
| **TMEM201** | 1.750980236 | 1.393084855 | 2.200821994 | 1.57E-06 |
| **ABCC5** | 1.908960234 | 1.465372344 | 2.486828136 | 1.65E-06 |
| **UPF3B** | 1.733750856 | 1.38404129 | 2.171822512 | 1.69E-06 |
| **SHCBP1** | 1.520504962 | 1.280790245 | 1.805085063 | 1.69E-06 |
| **MAST2** | 1.786174588 | 1.408236399 | 2.265542675 | 1.73E-06 |
| **CDC25B** | 1.478917588 | 1.259473901 | 1.736595914 | 1.80E-06 |
| **ECT2** | 1.407166298 | 1.22261137 | 1.619580056 | 1.92E-06 |
| **MAD2L1** | 1.509647002 | 1.274234668 | 1.7885513 | 1.92E-06 |
| **IGSF3** | 1.341244965 | 1.188475223 | 1.513652133 | 1.95E-06 |
| **MKI67** | 1.391543201 | 1.214429258 | 1.594487672 | 1.97E-06 |
| **CDK1** | 1.380795707 | 1.208823066 | 1.577233953 | 1.99E-06 |
| **CKAP2L** | 1.52420542 | 1.280804639 | 1.813861452 | 2.05E-06 |
| **H2AZ1** | 1.578651409 | 1.306994394 | 1.906771967 | 2.15E-06 |
| **YEATS2** | 1.776516054 | 1.400555904 | 2.253397584 | 2.17E-06 |
| **CDC45** | 1.446879899 | 1.241610134 | 1.686085981 | 2.22E-06 |
| **AC069544.2** | 1.863950156 | 1.440075866 | 2.412588298 | 2.24E-06 |
| **KIF11** | 1.456954634 | 1.246466365 | 1.702987632 | 2.28E-06 |
| **RNF145** | 1.539484593 | 1.287167694 | 1.841261883 | 2.31E-06 |
| **MTFR2** | 1.609768858 | 1.321211348 | 1.961348409 | 2.32E-06 |
| **COLGALT1** | 1.763202 | 1.393453056 | 2.231062813 | 2.32E-06 |
| **UBE2C** | 1.320943139 | 1.17677239 | 1.482776781 | 2.35E-06 |
| **CDC7** | 1.515986446 | 1.274925818 | 1.802626374 | 2.49E-06 |
| **CDCA5** | 1.421795215 | 1.227629331 | 1.646671013 | 2.63E-06 |
| **CDCA4** | 1.580856344 | 1.30579633 | 1.91385649 | 2.66E-06 |
| **POLQ** | 1.803776841 | 1.409606219 | 2.308170075 | 2.75E-06 |
| **PLCB3** | 1.854311898 | 1.432173639 | 2.400876905 | 2.80E-06 |
| **SMOX** | 1.393845175 | 1.213043053 | 1.601595564 | 2.81E-06 |
| **MACROH2A1** | 1.917096445 | 1.459928428 | 2.517423941 | 2.84E-06 |
| **PPT1** | 1.624321175 | 1.325486855 | 1.990528438 | 2.92E-06 |
| **XRCC2** | 1.657118767 | 1.340666356 | 2.048266964 | 2.99E-06 |
| **TACC3** | 1.462495931 | 1.246877572 | 1.71540045 | 2.99E-06 |
| **BUB1B** | 1.448745627 | 1.239750029 | 1.692973456 | 3.11E-06 |
| **GTF3C2** | 1.939810291 | 1.467889422 | 2.563451925 | 3.18E-06 |
| **KMT5A** | 2.000603812 | 1.493881501 | 2.679205553 | 3.26E-06 |
| **DR1** | 1.792770335 | 1.401964592 | 2.292515441 | 3.27E-06 |
| **EGLN3** | 1.303896002 | 1.166004581 | 1.45809443 | 3.27E-06 |
| **CNOT9** | 1.876117974 | 1.439157799 | 2.445748932 | 3.30E-06 |
| **E2F6** | 1.969231376 | 1.479854241 | 2.620442003 | 3.34E-06 |
| **POLA1** | 1.901166039 | 1.449702369 | 2.493223702 | 3.41E-06 |
| **CBX3** | 1.856069567 | 1.429560431 | 2.409827639 | 3.44E-06 |
| **RBL1** | 1.626629091 | 1.32393266 | 1.998532311 | 3.64E-06 |
| **NUP43** | 1.891264277 | 1.443788245 | 2.477427407 | 3.72E-06 |
| **RACGAP1** | 1.469433032 | 1.248135036 | 1.72996781 | 3.81E-06 |
| **ZMYM4** | 1.776950603 | 1.391521923 | 2.269136686 | 4.06E-06 |
| **TPGS2** | 1.615881369 | 1.317550428 | 1.981762933 | 4.06E-06 |
| **NCAPH** | 1.480022611 | 1.252611369 | 1.7487203 | 4.10E-06 |
| **NT5DC2** | 1.349425062 | 1.187857722 | 1.532968103 | 4.11E-06 |
| **CPSF6** | 1.885822707 | 1.439574236 | 2.470402147 | 4.13E-06 |
| **NASP** | 1.678598742 | 1.346242082 | 2.093006729 | 4.20E-06 |
| **ATP1B3** | 1.433617993 | 1.229324641 | 1.67186151 | 4.39E-06 |
| **GSR** | 1.565406446 | 1.292746621 | 1.895574353 | 4.44E-06 |
| **KIFC1** | 1.345373672 | 1.185237286 | 1.527145947 | 4.47E-06 |
| **STX3** | 1.516761203 | 1.268251593 | 1.813965432 | 5.05E-06 |
| **COMMD8** | 1.724906877 | 1.364607988 | 2.180335861 | 5.11E-06 |
| **CENPM** | 1.339070587 | 1.180830044 | 1.518516611 | 5.35E-06 |
| **PHF19** | 1.518188662 | 1.268116294 | 1.817575268 | 5.45E-06 |
| **RNF2** | 1.68359653 | 1.344433091 | 2.108321563 | 5.66E-06 |
| **DAB2** | 1.380390514 | 1.200944475 | 1.586649517 | 5.70E-06 |
| **CDC6** | 1.372566977 | 1.196859223 | 1.574069925 | 5.87E-06 |
| **TMEM164** | 1.562357078 | 1.288061164 | 1.895065007 | 5.90E-06 |
| **SMARCD1** | 1.74609712 | 1.371882425 | 2.222388082 | 5.92E-06 |
| **AC026401.3** | 1.504243844 | 1.260252962 | 1.795472506 | 6.14E-06 |
| **CDK16** | 1.623256357 | 1.315665274 | 2.002759556 | 6.20E-06 |
| **CFHR4** | 0.822366701 | 0.755342482 | 0.895338217 | 6.52E-06 |
| **MARCKSL1** | 1.37288603 | 1.196065357 | 1.575847039 | 6.64E-06 |
| **KIF2A** | 1.870302443 | 1.42432819 | 2.455916588 | 6.64E-06 |
| **DEPDC1B** | 1.347569119 | 1.183486796 | 1.534400331 | 6.70E-06 |
| **MTHFD1L** | 1.565526781 | 1.287946031 | 1.9029323 | 6.76E-06 |
| **CAP1** | 1.905917137 | 1.438865523 | 2.524572363 | 6.90E-06 |
| **CDT1** | 1.341700888 | 1.180329269 | 1.525134824 | 6.93E-06 |
| **MASTL** | 1.7127443 | 1.35438281 | 2.165926072 | 7.03E-06 |
| **ADH4** | 0.888454048 | 0.84377232 | 0.935501884 | 7.04E-06 |
| **RCC1** | 1.657681233 | 1.329389528 | 2.067044319 | 7.17E-06 |
| **PRKCD** | 1.533123301 | 1.272079233 | 1.847736364 | 7.23E-06 |
| **TROAP** | 1.38131557 | 1.199310732 | 1.590941075 | 7.42E-06 |
| **FOXD2-AS1** | 1.467547868 | 1.240766112 | 1.735779794 | 7.51E-06 |
| **MTF2** | 1.694051617 | 1.344784624 | 2.13403011 | 7.65E-06 |
| **SAE1** | 1.698777122 | 1.346153466 | 2.143770219 | 8.04E-06 |
| **RIBC2** | 1.424501122 | 1.219470756 | 1.66400337 | 8.10E-06 |
| **AGFG1** | 1.775864804 | 1.379408144 | 2.286267351 | 8.37E-06 |
| **GPD2** | 1.614392494 | 1.307456698 | 1.993383896 | 8.52E-06 |
| **PLBD1** | 1.290887383 | 1.153548071 | 1.44457806 | 8.63E-06 |
| **PSMC3IP** | 1.696829603 | 1.344205201 | 2.141957717 | 8.64E-06 |
| **TUBA1C** | 1.491247962 | 1.24990335 | 1.779193956 | 9.15E-06 |
| **CENPW** | 1.389061176 | 1.201342342 | 1.606112499 | 9.15E-06 |
| **CNOT6** | 1.710260249 | 1.34922896 | 2.167897521 | 9.17E-06 |
| **DKC1** | 1.71058399 | 1.349049817 | 2.169006326 | 9.36E-06 |
| **BARD1** | 1.664744085 | 1.328690964 | 2.085791915 | 9.41E-06 |
| **WASF1** | 1.473653043 | 1.241242478 | 1.749580222 | 9.52E-06 |
| **SNRNP40** | 1.821436026 | 1.396812682 | 2.375142522 | 9.53E-06 |
| **NAA40** | 1.655270853 | 1.323804463 | 2.069732861 | 9.85E-06 |
| **MPZL1** | 1.556181872 | 1.278987262 | 1.893452804 | 9.94E-06 |
| **NUP205** | 1.659827374 | 1.325643418 | 2.078256393 | 9.98E-06 |
| **FOXM1** | 1.336470726 | 1.175058791 | 1.52005501 | 1.00E-05 |
| **RAB10** | 1.674384569 | 1.331922724 | 2.104899658 | 1.01E-05 |
| **PCLAF** | 1.479506456 | 1.243080499 | 1.760899117 | 1.04E-05 |
| **PACC1** | 1.7205727 | 1.351480041 | 2.190465509 | 1.06E-05 |
| **AC107959.3** | 1.466088029 | 1.236307009 | 1.738576335 | 1.09E-05 |
| **RFC4** | 1.497169295 | 1.250761876 | 1.792120419 | 1.09E-05 |
| **SMYD5** | 1.736744516 | 1.358029197 | 2.221072656 | 1.09E-05 |
| **LARP4B** | 1.78670683 | 1.379364848 | 2.314341489 | 1.10E-05 |
| **MED22** | 1.73614691 | 1.35703319 | 2.221173452 | 1.14E-05 |
| **IKBIP** | 1.595021803 | 1.294487309 | 1.965329853 | 1.17E-05 |
| **NCAPD2** | 1.468745603 | 1.236461309 | 1.744667327 | 1.21E-05 |
| **IARS1** | 1.619855837 | 1.304190399 | 2.011924743 | 1.29E-05 |
| **SLC39A10** | 1.502548896 | 1.251212079 | 1.804372914 | 1.30E-05 |
| **NUP62** | 1.708015322 | 1.34254435 | 2.172975769 | 1.31E-05 |
| **FOXK1** | 1.656407065 | 1.319736183 | 2.078964264 | 1.34E-05 |
| **TPM3** | 1.766352932 | 1.367003622 | 2.282366068 | 1.36E-05 |
| **GPRIN1** | 1.508887753 | 1.253560129 | 1.816221016 | 1.37E-05 |
| **FUBP1** | 1.637501461 | 1.310971184 | 2.045362299 | 1.39E-05 |
| **NAP1L1** | 1.542447997 | 1.26833514 | 1.875802182 | 1.42E-05 |
| **NDRG1** | 1.3293891 | 1.168468497 | 1.512471568 | 1.53E-05 |
| **GNAI3** | 1.87545053 | 1.410339068 | 2.493949698 | 1.53E-05 |
| **UHRF1** | 1.362433101 | 1.184100579 | 1.567623552 | 1.55E-05 |
| **DSCC1** | 1.571135903 | 1.279722564 | 1.928908729 | 1.59E-05 |
| **FHL3** | 1.538302174 | 1.264931305 | 1.87075264 | 1.60E-05 |
| **MAPK7** | 1.756039633 | 1.358777865 | 2.269447619 | 1.69E-05 |
| **TRIM28** | 1.622858659 | 1.301296603 | 2.023881582 | 1.73E-05 |
| **PLXNA1** | 1.455524992 | 1.226469398 | 1.72735904 | 1.73E-05 |
| **PRXL2B** | 1.503226096 | 1.247473687 | 1.811411913 | 1.84E-05 |
| **STIL** | 1.53528112 | 1.26129362 | 1.868786206 | 1.92E-05 |
| **LMNB1** | 1.364123059 | 1.183089099 | 1.572858478 | 1.92E-05 |
| **RAD51** | 1.468757277 | 1.231381215 | 1.751892844 | 1.92E-05 |
| **DROSHA** | 1.700986824 | 1.3330222 | 2.170523623 | 1.94E-05 |
| **MAP7D1** | 1.567552355 | 1.275353794 | 1.926697045 | 1.95E-05 |
| **SLC36A1** | 1.626367686 | 1.300676756 | 2.033611994 | 1.99E-05 |
| **NARS1** | 1.819253114 | 1.381707564 | 2.395356282 | 2.01E-05 |
| **FANCD2** | 1.540992379 | 1.262898691 | 1.880323046 | 2.06E-05 |
| **CENPF** | 1.34658012 | 1.174214488 | 1.544247698 | 2.06E-05 |
| **CHAF1B** | 1.392318605 | 1.19553737 | 1.621489337 | 2.07E-05 |
| **NUP107** | 1.705348616 | 1.333180393 | 2.181410646 | 2.15E-05 |
| **C4orf46** | 1.636816269 | 1.303232443 | 2.055786375 | 2.26E-05 |
| **MTA2** | 1.952960796 | 1.432545704 | 2.662432243 | 2.30E-05 |
| **PLK4** | 1.532675665 | 1.25768568 | 1.867791555 | 2.31E-05 |
| **HMGA1** | 1.344804313 | 1.172354432 | 1.542621062 | 2.33E-05 |
| **TOR4A** | 1.381095141 | 1.188860116 | 1.604413978 | 2.42E-05 |
| **H2AX** | 1.430679451 | 1.211365161 | 1.689699984 | 2.46E-05 |
| **RNF19B** | 1.560818743 | 1.26908378 | 1.919617277 | 2.47E-05 |
| **WDR62** | 1.479399745 | 1.233112813 | 1.774877027 | 2.49E-05 |
| **UBAP2** | 1.701066243 | 1.328674529 | 2.177829334 | 2.51E-05 |
| **KIF14** | 1.483668875 | 1.234620649 | 1.78295522 | 2.58E-05 |
| **NECTIN1** | 1.494527874 | 1.239290315 | 1.802332786 | 2.61E-05 |
| **SLC25A24** | 1.3872583 | 1.190355932 | 1.616731215 | 2.78E-05 |
| **LAPTM4B** | 1.316378639 | 1.157581846 | 1.496959136 | 2.78E-05 |
| **VPS35** | 1.73020794 | 1.338336302 | 2.236821575 | 2.86E-05 |
| **ANXA5** | 1.414633523 | 1.202130462 | 1.664701186 | 2.96E-05 |
| **LRP10** | 1.568105667 | 1.269436088 | 1.937045438 | 3.01E-05 |
| **ZNF207** | 1.916184448 | 1.411694954 | 2.600960516 | 3.02E-05 |
| **MCM4** | 1.41581459 | 1.202394453 | 1.667115937 | 3.03E-05 |
| **SNX7** | 1.376282733 | 1.184408096 | 1.599241148 | 3.06E-05 |
| **TOP2A** | 1.267115577 | 1.133614591 | 1.416338408 | 3.08E-05 |
| **AURKB** | 1.313359095 | 1.154908663 | 1.493548508 | 3.25E-05 |
| **MSC** | 1.181646559 | 1.092154536 | 1.278471631 | 3.27E-05 |
| **SLC38A1** | 1.270659307 | 1.134853741 | 1.42271644 | 3.27E-05 |
| **MCM2** | 1.307615767 | 1.152126053 | 1.484090209 | 3.29E-05 |
| **RAP1GAP** | 1.287729457 | 1.142675832 | 1.451196487 | 3.36E-05 |
| **ARHGAP11A** | 1.44975602 | 1.216226553 | 1.728125827 | 3.41E-05 |
| **PHF21A** | 1.653785617 | 1.303538716 | 2.098140113 | 3.43E-05 |
| **PIGS** | 1.481600684 | 1.229997589 | 1.784670642 | 3.47E-05 |
| **DCLRE1B** | 1.689836203 | 1.318041622 | 2.166506995 | 3.50E-05 |
| **RNF34** | 1.716435403 | 1.328528074 | 2.217604996 | 3.58E-05 |
| **LRRC42** | 1.59320381 | 1.277247976 | 1.987318383 | 3.63E-05 |
| **RAD51AP1** | 1.389375166 | 1.188311926 | 1.624458451 | 3.74E-05 |
| **SET** | 1.759783268 | 1.344289591 | 2.303697931 | 3.90E-05 |
| **PKM** | 1.22655162 | 1.112667276 | 1.352092319 | 4.00E-05 |
| **RHNO1** | 1.548229489 | 1.256540732 | 1.907629804 | 4.06E-05 |
| **STARD3NL** | 1.597210895 | 1.277086194 | 1.99758063 | 4.08E-05 |
| **ASAP1** | 1.467843008 | 1.221659425 | 1.763636455 | 4.18E-05 |
| **CDK4** | 1.459219862 | 1.217899438 | 1.74835667 | 4.18E-05 |
| **CENPK** | 1.512035218 | 1.240325752 | 1.843266172 | 4.29E-05 |
| **VPS37C** | 1.762554622 | 1.343384808 | 2.312515951 | 4.31E-05 |
| **GPD1L** | 1.404817075 | 1.193559219 | 1.653467194 | 4.35E-05 |
| **ICMT** | 1.713225655 | 1.323418547 | 2.217848733 | 4.36E-05 |
| **XPR1** | 1.546397198 | 1.254408967 | 1.906351402 | 4.45E-05 |
| **QSER1** | 1.663862333 | 1.303004473 | 2.124657221 | 4.47E-05 |
| **LRRC41** | 1.778026637 | 1.348736276 | 2.343956174 | 4.47E-05 |
| **TBC1D10B** | 1.742285725 | 1.334517045 | 2.274650263 | 4.48E-05 |
| **YWHAZ** | 1.517131507 | 1.241842216 | 1.853446421 | 4.50E-05 |
| **FEN1** | 1.432251714 | 1.205143022 | 1.702158941 | 4.54E-05 |
| **CHML** | 1.391833058 | 1.187193569 | 1.631746761 | 4.61E-05 |
| **NDRG3** | 1.512197386 | 1.239413555 | 1.845018497 | 4.61E-05 |
| **ZIC2** | 1.294814665 | 1.143451473 | 1.466214401 | 4.63E-05 |
| **HAUS6** | 1.604095025 | 1.277760837 | 2.013773449 | 4.66E-05 |
| **CDCA7L** | 1.376330317 | 1.180147498 | 1.605125753 | 4.68E-05 |
| **RUSC1** | 1.518411263 | 1.241702355 | 1.856783757 | 4.72E-05 |
| **PON1** | 0.867204173 | 0.809658102 | 0.928840305 | 4.76E-05 |
| **PTMA** | 1.721027557 | 1.324807202 | 2.235748605 | 4.77E-05 |
| **AP003469.2** | 1.379555086 | 1.181271106 | 1.611122312 | 4.82E-05 |
| **ERI1** | 1.666744479 | 1.302430206 | 2.13296432 | 4.91E-05 |
| **TMEM65** | 1.445043024 | 1.209687326 | 1.726189318 | 4.94E-05 |
| **AKIRIN1** | 1.662988299 | 1.300696884 | 2.126191059 | 4.97E-05 |
| **DDX11** | 1.470244796 | 1.220392826 | 1.771249153 | 5.00E-05 |
| **PAQR4** | 1.385530441 | 1.183512552 | 1.622031468 | 5.01E-05 |
| **CASP2** | 1.569573557 | 1.262081802 | 1.951982152 | 5.07E-05 |
| **MARCKS** | 1.409524219 | 1.193748909 | 1.664301854 | 5.14E-05 |
| **DLGAP4** | 1.672660252 | 1.303594007 | 2.146214468 | 5.25E-05 |
| **ARPC2** | 1.759525602 | 1.337912766 | 2.314000152 | 5.28E-05 |
| **AC241585.1** | 1.558011336 | 1.256641239 | 1.931656586 | 5.28E-05 |
| **USP1** | 1.519455475 | 1.240515482 | 1.861117393 | 5.28E-05 |
| **SLC25A19** | 1.649813865 | 1.293603522 | 2.104111299 | 5.48E-05 |
| **STX6** | 1.65311198 | 1.294587458 | 2.110926692 | 5.58E-05 |
| **POGK** | 1.513190518 | 1.236943659 | 1.85113164 | 5.64E-05 |
| **DGKZ** | 1.822489817 | 1.36035799 | 2.441614015 | 5.76E-05 |
| **UBE2T** | 1.381053543 | 1.179978041 | 1.616393544 | 5.79E-05 |
| **ADAM9** | 1.377603899 | 1.178217696 | 1.610731624 | 5.92E-05 |
| **FANCI** | 1.464749061 | 1.215621292 | 1.764932734 | 6.00E-05 |
| **ABCC1** | 1.329436395 | 1.15680739 | 1.527826624 | 6.01E-05 |
| **B4GALT5** | 1.440515026 | 1.205262954 | 1.721685325 | 6.02E-05 |
| **PIF1** | 1.504797366 | 1.232317459 | 1.83752579 | 6.08E-05 |
| **CDKN2D** | 1.55844896 | 1.254437642 | 1.936137021 | 6.14E-05 |
| **CAPZA1** | 1.693726585 | 1.308725875 | 2.191986725 | 6.20E-05 |
| **NSD2** | 1.531186686 | 1.243009 | 1.886175134 | 6.21E-05 |
| **NPC1** | 1.533819945 | 1.244008377 | 1.891147735 | 6.24E-05 |
| **TMEM165** | 1.558874158 | 1.254157731 | 1.937626011 | 6.32E-05 |
| **TRIM45** | 1.377442078 | 1.177415597 | 1.611450265 | 6.33E-05 |
| **SLC22A15** | 1.349446638 | 1.165103099 | 1.562957158 | 6.36E-05 |
| **INTS8** | 1.61708474 | 1.27707593 | 2.047617528 | 6.59E-05 |
| **FAM219A** | 1.694896152 | 1.30798249 | 2.196262557 | 6.59E-05 |
| **TSEN54** | 1.569829738 | 1.257373941 | 1.959930396 | 6.82E-05 |
| **CCNB2** | 1.316459008 | 1.149768709 | 1.507315608 | 6.88E-05 |
| **KCTD17** | 1.270720017 | 1.128970591 | 1.430266985 | 7.18E-05 |
| **LIMK1** | 1.394136201 | 1.183036583 | 1.64290418 | 7.30E-05 |
| **WASF2** | 1.619425431 | 1.276130736 | 2.055070577 | 7.31E-05 |
| **AACS** | 1.53161098 | 1.240448586 | 1.891116021 | 7.40E-05 |
| **CORO1C** | 1.575809431 | 1.257996139 | 1.97391334 | 7.59E-05 |
| **YWHAQ** | 1.548476782 | 1.246394238 | 1.923773612 | 7.84E-05 |
| **DSG2** | 1.24452999 | 1.116310953 | 1.387476214 | 8.03E-05 |
| **PKMYT1** | 1.415505717 | 1.190921138 | 1.682442582 | 8.07E-05 |
| **FOXJ3** | 1.685946299 | 1.300055396 | 2.186379853 | 8.19E-05 |
| **TMEM184B** | 1.503458653 | 1.227059582 | 1.842117494 | 8.35E-05 |
| **LDHD** | 0.763749944 | 0.667746576 | 0.873555923 | 8.41E-05 |
| **ARHGEF2** | 1.394903763 | 1.181459893 | 1.646908642 | 8.57E-05 |
| **NDE1** | 1.635735223 | 1.279411502 | 2.091297221 | 8.65E-05 |
| **CLIC1** | 1.391667382 | 1.179830519 | 1.641539249 | 8.75E-05 |
| **CCDC112** | 1.525282181 | 1.235187667 | 1.883507902 | 8.77E-05 |
| **TMCO3** | 1.377164098 | 1.17337616 | 1.616345225 | 8.97E-05 |
| **RCC2** | 1.422708687 | 1.192578883 | 1.697246225 | 8.99E-05 |
| **PRKDC** | 1.486772496 | 1.218979847 | 1.813395406 | 9.07E-05 |
| **LHFPL2** | 1.399405555 | 1.182489813 | 1.656112288 | 9.21E-05 |
| **YBX1P10** | 1.606341766 | 1.266346786 | 2.037620263 | 9.38E-05 |
| **DYNC1H1** | 1.597031562 | 1.26223141 | 2.020635668 | 9.62E-05 |
| **TCF3** | 1.533416016 | 1.236961602 | 1.900919701 | 9.62E-05 |
| **S100PBP** | 1.712119575 | 1.306520642 | 2.243633469 | 9.69E-05 |
| **ASF1B** | 1.299966591 | 1.13930003 | 1.4832907 | 9.72E-05 |
| **TUBA4A** | 1.376911105 | 1.17221882 | 1.617346659 | 9.82E-05 |
| **TOPBP1** | 1.528997755 | 1.23479806 | 1.893292687 | 9.85E-05 |
| **TRAF3** | 1.614629668 | 1.267639236 | 2.056601667 | 0.000103985 |
| **PTGFRN** | 1.356748681 | 1.162942553 | 1.582852892 | 0.000104698 |
| **PJA1** | 1.504727195 | 1.223948607 | 1.849917487 | 0.000105438 |
| **TTF2** | 1.578633732 | 1.253121104 | 1.98870201 | 0.000106594 |
| **CALU** | 1.473490265 | 1.211082743 | 1.792754105 | 0.000107101 |
| **PNMA1** | 1.360858744 | 1.164369152 | 1.590506342 | 0.000107634 |
| **ME2** | 1.634411673 | 1.274259718 | 2.096355618 | 0.00010958 |
| **AGRN** | 1.314577097 | 1.14436363 | 1.510108237 | 0.000110649 |
| **NPAS2** | 1.496395242 | 1.21979654 | 1.835714929 | 0.000110929 |
| **CERS5** | 1.680264663 | 1.290977364 | 2.18693946 | 0.000113711 |
| **ANXA2P2** | 1.38848695 | 1.175063049 | 1.640674524 | 0.000115965 |
| **KNTC1** | 1.405622908 | 1.182017318 | 1.671528606 | 0.000117323 |
| **VANGL1** | 1.573596123 | 1.249050353 | 1.982469924 | 0.000119581 |
| **NT5C3A** | 1.539398651 | 1.235543464 | 1.917980446 | 0.000120395 |
| **CSF1** | 1.366096873 | 1.165124041 | 1.601735611 | 0.000121886 |
| **KHDRBS1** | 1.772736095 | 1.323299426 | 2.374816463 | 0.000124194 |
| **CCDC97** | 1.640982633 | 1.272751511 | 2.115749994 | 0.000133356 |
| **CAMSAP2** | 1.458769042 | 1.201260939 | 1.771477826 | 0.000138718 |
| **SLC10A1** | 0.888546695 | 0.836018982 | 0.944374763 | 0.000144229 |
| **KPNB1** | 1.587179182 | 1.250706021 | 2.014172568 | 0.000144522 |
| **GNB1** | 1.700002935 | 1.292725071 | 2.235595214 | 0.000146237 |
| **FAM102B** | 1.471433948 | 1.20471145 | 1.79720867 | 0.000153642 |
| **ARL6IP6** | 1.514010843 | 1.221297893 | 1.87687938 | 0.000154519 |
| **CAPRIN1** | 1.645181435 | 1.271081435 | 2.129385168 | 0.000155382 |
| **CBX1** | 1.485738224 | 1.210065477 | 1.824213741 | 0.00015629 |
| **NUDT1** | 1.354971252 | 1.15753018 | 1.586090043 | 0.000156509 |
| **HMGB2** | 1.35886656 | 1.159115933 | 1.593040243 | 0.00015671 |
| **B3GNT9** | 1.366541507 | 1.162104039 | 1.606943637 | 0.000158739 |
| **ASRGL1** | 1.320396813 | 1.142855448 | 1.525519039 | 0.000161713 |
| **CA9** | 1.128763187 | 1.059904317 | 1.202095616 | 0.000162239 |
| **PARD6B** | 1.451053439 | 1.195663295 | 1.760994163 | 0.000163772 |
| **POLD1** | 1.474813017 | 1.2048654 | 1.805241841 | 0.000165359 |
| **ZNF816** | 1.446419182 | 1.193380125 | 1.753111523 | 0.000168673 |
| **LAMB1** | 1.308230083 | 1.137336331 | 1.504801969 | 0.000168726 |
| **CDK2** | 1.489816872 | 1.210256457 | 1.833953703 | 0.000170112 |
| **UBE2Z** | 1.585902174 | 1.246417483 | 2.017851754 | 0.000175249 |
| **ADGRE5** | 1.315847613 | 1.140062004 | 1.51873752 | 0.000175712 |
| **DCK** | 1.470704854 | 1.202269177 | 1.79907529 | 0.000175792 |
| **INCENP** | 1.421541823 | 1.182757375 | 1.70853397 | 0.0001776 |
| **OBI1** | 1.604039477 | 1.252656123 | 2.053989595 | 0.000179965 |
| **CDCA7** | 1.261220603 | 1.116986585 | 1.424079241 | 0.000180068 |
| **CTHRC1** | 1.198952086 | 1.090346417 | 1.318375592 | 0.000180133 |
| **BAK1** | 1.4004145 | 1.173901119 | 1.670635406 | 0.000183259 |
| **CARMIL1** | 1.359283309 | 1.157349795 | 1.596450029 | 0.00018338 |
| **CCAR1** | 1.648978235 | 1.26829797 | 2.14391987 | 0.00018793 |
| **CLCF1** | 1.341098603 | 1.149550277 | 1.564564421 | 0.000189679 |
| **NEDD1** | 1.53312018 | 1.224906733 | 1.918886902 | 0.000190331 |
| **CFHR3** | 0.865742211 | 0.802579286 | 0.933876053 | 0.000191556 |
| **AL353572.4** | 1.252838558 | 1.112765773 | 1.410543432 | 0.000194331 |
| **CYRIB** | 1.464617669 | 1.198119134 | 1.790393674 | 0.000196176 |
| **MMD** | 1.335258037 | 1.14676607 | 1.554732103 | 0.000196294 |
| **CANT1** | 1.607833484 | 1.252227304 | 2.064424329 | 0.00019641 |
| **SMC4** | 1.314013091 | 1.137862324 | 1.517433495 | 0.000200311 |
| **SPIN4** | 1.498771606 | 1.210888281 | 1.855097918 | 0.000200535 |
| **IGF2BP3** | 1.327705805 | 1.143262176 | 1.541905909 | 0.000203655 |
| **GINS3** | 1.572941604 | 1.238312033 | 1.997998262 | 0.000206116 |
| **STRN4** | 1.661137264 | 1.269191536 | 2.174121819 | 0.00021899 |
| **IFT80** | 1.487798033 | 1.205087337 | 1.836832003 | 0.000219949 |
| **AGAP1** | 1.569454584 | 1.235652048 | 1.993431481 | 0.00022051 |
| **ANKLE2** | 1.707777536 | 1.285563033 | 2.268658974 | 0.00022113 |
| **GTPBP2** | 1.441812563 | 1.187297906 | 1.750886155 | 0.000222097 |
| **LGALS3** | 1.22803202 | 1.100925143 | 1.369813972 | 0.000228939 |
| **TRAF2** | 1.53026621 | 1.220184923 | 1.919147361 | 0.000231007 |
| **CCDC93** | 1.591960348 | 1.242872749 | 2.039096724 | 0.000231882 |
| **TFDP1** | 1.406873522 | 1.172960519 | 1.687433698 | 0.000233711 |
| **LINC00205** | 1.567068704 | 1.233470239 | 1.990890616 | 0.000235043 |
| **CREB1** | 1.728169951 | 1.290840555 | 2.313664044 | 0.000237941 |
| **RPE** | 1.671244434 | 1.270791149 | 2.197889055 | 0.000238233 |
| **PUDP** | 1.45145984 | 1.18986021 | 1.770574097 | 0.000238431 |
| **FAM110A** | 1.466343806 | 1.195474189 | 1.79858685 | 0.000239326 |
| **IQCB1** | 1.551719299 | 1.227442554 | 1.961666374 | 0.000239429 |
| **VNN2** | 1.191853866 | 1.085229354 | 1.308954307 | 0.000242086 |
| **ODC1** | 1.355327059 | 1.152057304 | 1.594461864 | 0.000245122 |
| **ITGA5** | 1.376376498 | 1.160320381 | 1.632663095 | 0.000245701 |
| **CDKN2B** | 1.397680822 | 1.168365635 | 1.672003713 | 0.000250457 |
| **MFAP2** | 1.315354283 | 1.135799189 | 1.523294705 | 0.000251803 |
| **TYRO3** | 1.277647897 | 1.120419491 | 1.456940157 | 0.000255159 |
| **NCOA5** | 1.599056131 | 1.243001071 | 2.057102419 | 0.000259603 |
| **KIAA1522** | 1.373160018 | 1.158146548 | 1.6280914 | 0.000262473 |
| **BTBD10** | 1.615971071 | 1.248784127 | 2.091124035 | 0.000262983 |
| **NEMP1** | 1.410934688 | 1.1727319 | 1.697520716 | 0.000263472 |
| **CAPG** | 1.22450121 | 1.098183493 | 1.365348525 | 0.000266391 |
| **MCM3** | 1.366435425 | 1.155236313 | 1.616245741 | 0.000267925 |
| **NUP188** | 1.539180676 | 1.220514205 | 1.941048408 | 0.000268851 |
| **CCDC6** | 1.602403501 | 1.242806604 | 2.0660471 | 0.000276454 |
| **ARHGAP1** | 1.549069241 | 1.22330265 | 1.961587768 | 0.000279982 |
| **FAM222B** | 1.542264052 | 1.220473431 | 1.948898145 | 0.000284873 |
| **ARF4** | 1.592246443 | 1.238314509 | 2.047338313 | 0.000287349 |
| **PTPN12** | 1.494381202 | 1.202721687 | 1.85676803 | 0.000287611 |
| **UBE2D1** | 1.564526606 | 1.228209193 | 1.992936964 | 0.000289413 |
| **SLC27A5** | 0.849043655 | 0.777097089 | 0.927651303 | 0.000291995 |
| **DNMT1** | 1.392997099 | 1.164223761 | 1.666725061 | 0.000293299 |
| **TRIM65** | 1.538237669 | 1.218159933 | 1.942417464 | 0.00029704 |
| **TLCD3A** | 1.335011396 | 1.14153636 | 1.561277845 | 0.000297914 |
| **CTDSPL** | 1.455623194 | 1.187158203 | 1.784799092 | 0.000307006 |
| **SCRN1** | 1.244737372 | 1.105214181 | 1.401874092 | 0.000307113 |
| **MAPRE1** | 1.444807443 | 1.183006392 | 1.76454545 | 0.000309048 |
| **GNA12** | 1.496168434 | 1.201947063 | 1.862411459 | 0.00031042 |
| **S100A11** | 1.222237045 | 1.095888203 | 1.363153093 | 0.000312572 |
| **PIP4K2A** | 1.482256475 | 1.196658278 | 1.836016428 | 0.000313354 |
| **MICALL1** | 1.505904171 | 1.205235742 | 1.881579919 | 0.000314879 |
| **ZNF611** | 1.543902405 | 1.218769642 | 1.955771258 | 0.000318521 |
| **ADAM15** | 1.425820852 | 1.175369099 | 1.72963974 | 0.00031887 |
| **CHPF2** | 1.576012314 | 1.230142984 | 2.019126919 | 0.000320096 |
| **TTF1** | 1.702031684 | 1.273852313 | 2.274134783 | 0.000321801 |
| **ATP2C1** | 1.585002425 | 1.232620146 | 2.038123989 | 0.000330433 |
| **RAB35** | 1.687041779 | 1.267948292 | 2.244657753 | 0.000331587 |
| **FIGNL1** | 1.480036175 | 1.194757799 | 1.833431915 | 0.000332254 |
| **ENOPH1** | 1.599713883 | 1.237615955 | 2.067753327 | 0.00033311 |
| **BAIAP2L2** | 1.20347734 | 1.08748435 | 1.331842346 | 0.000341151 |
| **ITGAV** | 1.286083874 | 1.120636388 | 1.475957545 | 0.000342201 |
| **ADAM17** | 1.539893806 | 1.215822778 | 1.950344223 | 0.000342394 |
| **SCMH1** | 1.486468998 | 1.196393931 | 1.846875034 | 0.000345118 |
| **IKBKE** | 1.326940375 | 1.136338138 | 1.549513038 | 0.000349629 |
| **ETV4** | 1.17243322 | 1.074472276 | 1.279325382 | 0.000352244 |
| **RRAS2** | 1.467244705 | 1.188702964 | 1.811055487 | 0.000357925 |
| **DYRK2** | 1.448767405 | 1.181925999 | 1.775853136 | 0.000358005 |
| **VRK2** | 1.496308343 | 1.199133114 | 1.867131038 | 0.000360307 |
| **ADH1B** | 0.879005557 | 0.818791514 | 0.943647748 | 0.000368056 |
| **DENND5A** | 1.560449258 | 1.221375627 | 1.993655214 | 0.000371161 |
| **NDOR1** | 1.533395027 | 1.211662087 | 1.940557796 | 0.000373834 |
| **SOGA1** | 1.432780791 | 1.175108918 | 1.746953634 | 0.000377722 |
| **DEGS1** | 1.437870742 | 1.17689833 | 1.756712724 | 0.0003795 |
| **UNC5B** | 1.306823713 | 1.127479208 | 1.514695974 | 0.000380779 |
| **PRTFDC1** | 1.370101269 | 1.151497294 | 1.630205731 | 0.000384424 |
| **DCAF16** | 1.423791839 | 1.170853781 | 1.731371784 | 0.000399263 |
| **CUEDC1** | 1.391377959 | 1.158341493 | 1.671296967 | 0.000413017 |
| **PRKCI** | 1.503278177 | 1.198395432 | 1.885725877 | 0.000423604 |
| **STK11IP** | 1.600806195 | 1.232134693 | 2.079789238 | 0.000426692 |
| **PDE7A** | 1.468977412 | 1.185588417 | 1.820104351 | 0.000436858 |
| **TULP3** | 1.494731053 | 1.194680376 | 1.870141141 | 0.000438259 |
| **CXCL8** | 1.151563352 | 1.064437206 | 1.245820934 | 0.000438681 |
| **RGS2** | 1.196844436 | 1.082543881 | 1.323213433 | 0.000450335 |
| **ZNF142** | 1.686831175 | 1.259150681 | 2.259776734 | 0.000457438 |
| **IFT52** | 1.464186708 | 1.182976279 | 1.81224489 | 0.000457933 |
| **MIS18BP1** | 1.473719842 | 1.186322671 | 1.830741522 | 0.00045891 |
| **OLFML2B** | 1.275922594 | 1.113308725 | 1.462288428 | 0.00045994 |
| **B4GALT4** | 1.596849769 | 1.228940714 | 2.07490008 | 0.000460306 |
| **KLC2** | 1.471299585 | 1.185381881 | 1.826181507 | 0.000460847 |
| **ACTR3** | 1.584232002 | 1.224487586 | 2.04966638 | 0.00046351 |
| **CHST1** | 1.329724573 | 1.133129566 | 1.560428297 | 0.000481051 |
| **BAZ1A** | 1.516515335 | 1.200274566 | 1.916077227 | 0.000483249 |
| **MED15** | 1.589749644 | 1.22516868 | 2.062821202 | 0.000486814 |
| **TREM2** | 1.22504806 | 1.092701997 | 1.373423636 | 0.000501786 |
| **CKAP4** | 1.350737957 | 1.140131146 | 1.600248388 | 0.000508323 |
| **ANKRD13D** | 1.462941731 | 1.180191506 | 1.813433242 | 0.000516807 |
| **TYMS** | 1.323428623 | 1.129584842 | 1.5505372 | 0.000524523 |
| **PAFAH1B3** | 1.238296805 | 1.09734836 | 1.397349313 | 0.000526892 |
| **TMEM43** | 1.481083124 | 1.186092767 | 1.849439843 | 0.000528335 |
| **UBE2Q2** | 1.334150652 | 1.133404187 | 1.570452961 | 0.000530077 |
| **ACTL6A** | 1.43917339 | 1.171295751 | 1.768315172 | 0.000531032 |
| **CBFB** | 1.499043927 | 1.191963231 | 1.885236589 | 0.000537329 |
| **GNAZ** | 1.228115811 | 1.093019269 | 1.379910207 | 0.000548557 |
| **GLS** | 1.300266597 | 1.120271949 | 1.509181074 | 0.000552505 |
| **STK39** | 1.23403835 | 1.09521657 | 1.39045618 | 0.000552942 |
| **MFSD10** | 1.290575979 | 1.116282643 | 1.492083003 | 0.000568903 |
| **SNHG1** | 1.40697598 | 1.158619617 | 1.708568868 | 0.000569427 |
| **DNMT3A** | 1.427430498 | 1.165624257 | 1.748040001 | 0.000576495 |
| **NCF2** | 1.255328924 | 1.102791658 | 1.428965025 | 0.000581224 |
| **BTBD3** | 1.393442087 | 1.153430631 | 1.683396295 | 0.000581874 |
| **CIZ1** | 1.565850381 | 1.212715114 | 2.02181649 | 0.000583793 |
| **EPS8L3** | 1.17896552 | 1.072946941 | 1.295459863 | 0.000615973 |
| **DNM1L** | 1.584851306 | 1.217599447 | 2.062873524 | 0.000617526 |
| **NCBP1** | 1.685146309 | 1.249586431 | 2.272526343 | 0.000625473 |
| **RBM12** | 1.584088322 | 1.216828289 | 2.062193848 | 0.000630261 |
| **ARMCX1** | 1.306205182 | 1.120652597 | 1.522480724 | 0.000632817 |
| **DNAJC13** | 1.558768084 | 1.208228157 | 2.0110092 | 0.000637087 |
| **LRRC1** | 1.277422358 | 1.109745869 | 1.470433841 | 0.000648718 |
| **TTYH3** | 1.291893352 | 1.11502633 | 1.496815267 | 0.000651135 |
| **FBXL19** | 1.494801476 | 1.186256107 | 1.883599537 | 0.000654481 |
| **RFWD3** | 1.491911894 | 1.184471417 | 1.879151381 | 0.000679111 |
| **SENP1** | 1.587655024 | 1.215760539 | 2.073309992 | 0.000687004 |
| **PHF6** | 1.508963133 | 1.189600123 | 1.91406313 | 0.000696646 |
| **SPRED1** | 1.369473785 | 1.141833853 | 1.642496799 | 0.000699019 |
| **CD24** | 1.144658153 | 1.058551987 | 1.237768484 | 0.000709067 |
| **GRN** | 1.469798745 | 1.175803408 | 1.837304039 | 0.000718918 |
| **RBM38** | 1.432725819 | 1.163081013 | 1.764884173 | 0.000724728 |
| **TMEM45A** | 1.185382812 | 1.074021842 | 1.30829035 | 0.000728411 |
| **EPB41L2** | 1.353984568 | 1.135327936 | 1.614753018 | 0.000745331 |
| **DAGLA** | 1.334141442 | 1.128217226 | 1.577651313 | 0.000750771 |
| **RDH16** | 0.880634367 | 0.817867669 | 0.948218052 | 0.000753455 |
| **PAG1** | 1.329707472 | 1.126383589 | 1.569733417 | 0.000763796 |
| **KRBA1** | 1.34576782 | 1.131994935 | 1.599910891 | 0.000766213 |
| **ZNF124** | 1.522820219 | 1.191730608 | 1.945893983 | 0.000772991 |
| **TTLL4** | 1.29271687 | 1.112939015 | 1.501535021 | 0.000777908 |
| **UBA2** | 1.477448285 | 1.176400218 | 1.855536409 | 0.000786818 |
| **HCFC1** | 1.530140779 | 1.193287758 | 1.962083989 | 0.000799698 |
| **NFKBIE** | 1.332148808 | 1.12649368 | 1.575348781 | 0.000801787 |
| **STK4** | 1.560019647 | 1.20261703 | 2.023637815 | 0.00080892 |
| **FRMD8** | 1.575228454 | 1.207443701 | 2.055039653 | 0.000809647 |
| **PIMREG** | 1.300570567 | 1.11500836 | 1.517014454 | 0.000819914 |
| **WDR45B** | 1.53069156 | 1.191934316 | 1.965726317 | 0.000850899 |
| **WDR54** | 1.315632151 | 1.119631894 | 1.545943775 | 0.000859506 |
| **IL4I1** | 1.225793505 | 1.087456817 | 1.38172817 | 0.000861462 |
| **ENAH** | 1.320686344 | 1.121311273 | 1.555511356 | 0.000864594 |
| **RGS19** | 1.402877513 | 1.149363801 | 1.712308423 | 0.000872028 |
| **SMARCA4** | 1.500608096 | 1.181560244 | 1.905806046 | 0.000874926 |
| **RPRD1A** | 1.518507748 | 1.187201126 | 1.942270546 | 0.00087967 |
| **ENO2** | 1.221322547 | 1.085447426 | 1.374206366 | 0.000892103 |
| **PGK1** | 1.382160271 | 1.141928486 | 1.672930518 | 0.000892556 |
| **FUNDC1** | 1.496669389 | 1.179630528 | 1.898915979 | 0.000899575 |
| **NCAPD3** | 1.504257276 | 1.182054011 | 1.914286429 | 0.000900438 |
| **XRN2** | 1.541507541 | 1.193779451 | 1.990523038 | 0.000906727 |
| **SEPTIN2** | 1.406790412 | 1.149736948 | 1.721314833 | 0.000915391 |
| **CDK19** | 1.402480046 | 1.14825009 | 1.712998149 | 0.00091754 |
| **MMP14** | 1.229554138 | 1.087956644 | 1.389580538 | 0.000931594 |
| **DDX39A** | 1.406234343 | 1.149130335 | 1.720862263 | 0.000935276 |
| **ARNTL2** | 1.30109839 | 1.113075288 | 1.520882763 | 0.000949147 |
| **BRI3BP** | 1.44057122 | 1.159954507 | 1.789074853 | 0.000959082 |
| **STXBP5** | 1.497980828 | 1.178511906 | 1.904050821 | 0.000959683 |
| **ANKRD52** | 1.415785896 | 1.15177796 | 1.740309134 | 0.000960154 |
| **CDH24** | 1.396075296 | 1.145118266 | 1.702030517 | 0.000965925 |
| **RNF144A** | 1.351230971 | 1.130032334 | 1.615728225 | 0.000966072 |
| **PAPSS1** | 1.398383461 | 1.145720089 | 1.706766184 | 0.000974251 |
| **ADH1C** | 0.908450872 | 0.858061986 | 0.961798798 | 0.000974595 |
| **MEX3C** | 1.460104752 | 1.165644987 | 1.82894956 | 0.000988616 |
| **RAP2B** | 1.461890733 | 1.16621812 | 1.832525561 | 0.000988793 |
| **KDELR3** | 1.227540311 | 1.086476572 | 1.386919197 | 0.000996098 |
| **SELENON** | 1.440343922 | 1.159063633 | 1.789885002 | 0.000996334 |
| **TRIM32** | 1.525926376 | 1.186154845 | 1.963024739 | 0.001007785 |
| **C15orf48** | 1.206047494 | 1.078564803 | 1.348598206 | 0.001013194 |
| **PNP** | 1.403493129 | 1.146626602 | 1.717902725 | 0.001013969 |
| **PLXNA3** | 1.355906618 | 1.130595148 | 1.626119448 | 0.001024205 |
| **FZD1** | 1.268770536 | 1.100682856 | 1.462527254 | 0.001027236 |
| **NRM** | 1.264418423 | 1.099004479 | 1.454729238 | 0.001039359 |
| **CDYL** | 1.544377183 | 1.191073548 | 2.002479937 | 0.001040772 |
| **FSCN1** | 1.27240272 | 1.101553685 | 1.469750139 | 0.001057608 |
| **SOX12** | 1.296969299 | 1.109927916 | 1.515530277 | 0.001065849 |
| **PDCL** | 1.604795291 | 1.208543493 | 2.130968344 | 0.001078859 |
| **CDKN2A** | 1.223155823 | 1.083952247 | 1.380236234 | 0.001084283 |
| **GORAB** | 1.372658146 | 1.135142225 | 1.659871639 | 0.0010846 |
| **CAND1** | 1.532698657 | 1.186111566 | 1.980560042 | 0.001094914 |
| **ILF3** | 1.516651519 | 1.180815754 | 1.948002321 | 0.001108508 |
| **KITLG** | 1.336222008 | 1.122606856 | 1.590484902 | 0.001109172 |
| **FAAP24** | 1.486518169 | 1.171065396 | 1.886945234 | 0.001123752 |
| **ZIC5** | 1.315125191 | 1.115167399 | 1.550936899 | 0.001132518 |
| **SLC10A3** | 1.352829123 | 1.127765379 | 1.622807961 | 0.001133534 |
| **RLF** | 1.490384245 | 1.171667952 | 1.89579752 | 0.001151962 |
| **STK26** | 1.251250533 | 1.093010064 | 1.432400257 | 0.001157448 |
| **E2F3** | 1.351619257 | 1.126839521 | 1.621237613 | 0.001167226 |
| **RCAN3** | 1.389760721 | 1.139229797 | 1.695386539 | 0.001173518 |
| **IER3** | 1.198426054 | 1.074097783 | 1.337145489 | 0.001199113 |
| **SFXN3** | 1.281684765 | 1.102850887 | 1.489517627 | 0.001208818 |
| **PI4KB** | 1.486626223 | 1.169227458 | 1.890186133 | 0.001212764 |
| **VEGFA** | 1.384594664 | 1.136773222 | 1.686442244 | 0.001220743 |
| **FRAS1** | 1.23692658 | 1.087235279 | 1.407227482 | 0.001234435 |
| **CEP170** | 1.453832991 | 1.158352626 | 1.824686472 | 0.001246422 |
| **METTL9** | 1.435391992 | 1.152504129 | 1.787716086 | 0.001249441 |
| **RASA3** | 1.294236543 | 1.106592029 | 1.513699887 | 0.001249513 |
| **PTK7** | 1.230356961 | 1.08475947 | 1.39549669 | 0.001255088 |
| **ZBED5** | 1.489943879 | 1.169377643 | 1.898388236 | 0.001256051 |
| **SPATS2L** | 1.450132103 | 1.156973532 | 1.817572364 | 0.001258307 |
| **VEZF1** | 1.444248025 | 1.155002152 | 1.805929411 | 0.001265319 |
| **PLA2G7** | 1.237761565 | 1.087100937 | 1.409302153 | 0.001276914 |
| **ZNF532** | 1.353966324 | 1.125966532 | 1.628134366 | 0.001277368 |
| **ZBTB12** | 1.315467551 | 1.113162962 | 1.554538676 | 0.001289703 |
| **POGLUT3** | 1.321675769 | 1.11505352 | 1.566585644 | 0.001302012 |
| **AL355488.1** | 1.385583678 | 1.135728604 | 1.690405721 | 0.001306894 |
| **CSNK2A1** | 1.562478325 | 1.190229619 | 2.051149187 | 0.001307936 |
| **GPR107** | 1.487046544 | 1.167441537 | 1.894148318 | 0.001309321 |
| **SLC26A2** | 1.423416492 | 1.147128498 | 1.766248953 | 0.001342993 |
| **RBBP8** | 1.361758458 | 1.127432385 | 1.644786971 | 0.001351161 |
| **SGCB** | 1.260422654 | 1.094021152 | 1.45213396 | 0.001355889 |
| **ZFC3H1** | 1.474532166 | 1.161785589 | 1.871468478 | 0.001408477 |
| **MARK2** | 1.508755163 | 1.172006941 | 1.942259948 | 0.001414681 |
| **LLGL1** | 1.401580478 | 1.138998618 | 1.724697296 | 0.001424722 |
| **PYGB** | 1.314311393 | 1.111036308 | 1.554777666 | 0.001431525 |
| **DBN1** | 1.217256683 | 1.078668508 | 1.373650774 | 0.001433118 |
| **CHMP3** | 1.409754711 | 1.140983311 | 1.741838226 | 0.001462403 |
| **BICDL1** | 1.269105093 | 1.095811919 | 1.469803083 | 0.001465612 |
| **ARMCX6** | 1.29268897 | 1.103581997 | 1.514200828 | 0.001465965 |
| **HMGXB4** | 1.476294431 | 1.16113503 | 1.876995518 | 0.001476168 |
| **NOL4L** | 1.48380885 | 1.162549318 | 1.893845423 | 0.001525351 |
| **CERS6** | 1.342799666 | 1.119013042 | 1.611340419 | 0.001530415 |
| **PHTF2** | 1.498951639 | 1.166881122 | 1.925522638 | 0.001535763 |
| **TASOR2** | 1.406684138 | 1.138458236 | 1.738105272 | 0.001570587 |
| **UHRF1BP1** | 1.440844477 | 1.148777 | 1.807167801 | 0.001577764 |
| **DTNBP1** | 1.411011941 | 1.139593965 | 1.747073747 | 0.001584227 |
| **PFKFB3** | 1.18558085 | 1.066499689 | 1.317958145 | 0.001621127 |
| **INAVA** | 1.19845041 | 1.070846625 | 1.341259665 | 0.001623562 |
| **CD109** | 1.247908529 | 1.087268475 | 1.432282581 | 0.001632708 |
| **BCL9** | 1.303213719 | 1.105039113 | 1.536928401 | 0.001650987 |
| **PEA15** | 1.407709322 | 1.137177131 | 1.742600587 | 0.001686394 |
| **POLD3** | 1.410555147 | 1.137947531 | 1.748468859 | 0.001693306 |
| **ZBTB41** | 1.368577729 | 1.124477894 | 1.66566636 | 0.00174554 |
| **SOAT1** | 1.405590165 | 1.135722366 | 1.739583344 | 0.001747941 |
| **SP4** | 1.474555255 | 1.156060206 | 1.880795817 | 0.001759964 |
| **CHST11** | 1.253718813 | 1.087985965 | 1.444697737 | 0.001774022 |
| **SPRING1** | 1.301877359 | 1.103309851 | 1.536181932 | 0.001782141 |
| **SH3PXD2B** | 1.341641166 | 1.115635828 | 1.613430633 | 0.001792691 |
| **NSMAF** | 1.419294803 | 1.139122632 | 1.768376541 | 0.001802721 |
| **STXBP3** | 1.46070495 | 1.1512542 | 1.853334346 | 0.001811137 |
| **TUBA1B** | 1.293110809 | 1.100228165 | 1.519807998 | 0.001815109 |
| **ZNF738** | 1.410412038 | 1.136175813 | 1.750840049 | 0.001825364 |
| **C11orf80** | 1.377529897 | 1.126047119 | 1.685176923 | 0.001844299 |
| **ANG** | 0.836907807 | 0.74818919 | 0.936146481 | 0.001845365 |
| **SLC39A6** | 1.314309985 | 1.106585723 | 1.561027493 | 0.001846705 |
| **PRPF38B** | 1.450619467 | 1.14767382 | 1.833532143 | 0.001855794 |
| **FCER1G** | 1.253126222 | 1.087130067 | 1.444468675 | 0.001856767 |
| **ZNF320** | 1.293907655 | 1.100074417 | 1.521894331 | 0.001859241 |
| **NPEPPS** | 1.407504096 | 1.134471439 | 1.746247383 | 0.001892252 |
| **SOX4** | 1.18924684 | 1.066039996 | 1.326693231 | 0.001896359 |
| **ZBTB26** | 1.518025307 | 1.166398242 | 1.97565527 | 0.001903437 |
| **RYK** | 1.541667937 | 1.172997408 | 2.026210811 | 0.001907616 |
| **TEX10** | 1.553633548 | 1.175884876 | 2.052732585 | 0.00193593 |
| **ACSM2A** | 0.870679288 | 0.797671183 | 0.950369575 | 0.001940474 |
| **IER5** | 1.406710713 | 1.133607891 | 1.745608023 | 0.001943862 |
| **SEPTIN9** | 1.512260783 | 1.163777651 | 1.96509417 | 0.001969008 |
| **HNRNPH3** | 1.529747715 | 1.168319694 | 2.002986068 | 0.0019936 |
| **FAM241B** | 1.273067849 | 1.092224925 | 1.483853474 | 0.002011888 |
| **ELF4** | 1.256019056 | 1.086591307 | 1.451864983 | 0.002047518 |
| **MRE11** | 1.528505731 | 1.167076916 | 2.001864435 | 0.00205342 |
| **MFSD14B** | 1.425448016 | 1.137726012 | 1.785932663 | 0.002058325 |
| **SDC3** | 1.332315918 | 1.109782553 | 1.599471628 | 0.002090401 |
| **RBBP4** | 1.365261127 | 1.119488088 | 1.664991317 | 0.002107935 |
| **HNRNPL** | 1.577136554 | 1.179286516 | 2.109207285 | 0.002127606 |
| **NEURL3** | 1.192172218 | 1.065622039 | 1.333751128 | 0.00214018 |
| **COLCA2** | 1.245402064 | 1.082578789 | 1.432714473 | 0.002141337 |
| **SCPEP1** | 1.223430956 | 1.075548909 | 1.391645969 | 0.002154948 |
| **ANKRD27** | 1.374936248 | 1.121703556 | 1.685338052 | 0.002171013 |
| **DHX9** | 1.440305368 | 1.140340665 | 1.819175285 | 0.00219738 |
| **HIF1A** | 1.257289221 | 1.085835389 | 1.45581568 | 0.002206934 |
| **SMC2** | 1.353930674 | 1.115135683 | 1.643861188 | 0.002207939 |
| **SUCO** | 1.3594679 | 1.116747519 | 1.654942536 | 0.002210635 |
| **TAT** | 0.912237638 | 0.860096274 | 0.967539952 | 0.002221916 |
| **DNAJC10** | 1.402744824 | 1.129263387 | 1.742457132 | 0.002223464 |
| **XXYLT1** | 1.41447363 | 1.13257701 | 1.76653387 | 0.002229692 |
| **KANSL2** | 1.502959041 | 1.157005382 | 1.95235555 | 0.002268764 |
| **C6** | 0.870588347 | 0.796457759 | 0.951618666 | 0.002272333 |
| **UXS1** | 1.454136494 | 1.143044426 | 1.849895676 | 0.002299591 |
| **MGAT4A** | 1.38941465 | 1.124353731 | 1.71696239 | 0.002324969 |
| **IVNS1ABP** | 1.341468141 | 1.110257173 | 1.620828774 | 0.002337605 |
| **NCK2** | 1.213874273 | 1.071340481 | 1.375371115 | 0.002355808 |
| **MIR4292** | 1.279176145 | 1.091398815 | 1.499260936 | 0.002367931 |
| **GPT** | 0.841611684 | 0.753029685 | 0.940613951 | 0.002374367 |
| **KDM5C** | 1.425522846 | 1.13400288 | 1.791984326 | 0.002387265 |
| **GPX7** | 1.200520967 | 1.066969464 | 1.350788977 | 0.002387363 |
| **RASSF3** | 1.271089429 | 1.088783572 | 1.483920567 | 0.002390622 |
| **SLC41A1** | 1.278010672 | 1.09064237 | 1.497568152 | 0.002424238 |
| **CBL** | 1.437890371 | 1.137055197 | 1.818318693 | 0.002426083 |
| **IGFBP3** | 1.203374405 | 1.067648992 | 1.35635398 | 0.002428978 |
| **CASP8** | 1.487430665 | 1.150507053 | 1.92302166 | 0.002446915 |
| **TDG** | 1.453597388 | 1.141083868 | 1.851700323 | 0.002457027 |
| **SKAP2** | 1.312173652 | 1.100476618 | 1.564594526 | 0.002473775 |
| **C12orf75** | 1.150906022 | 1.050708189 | 1.26065894 | 0.002491791 |
| **SERPINH1** | 1.280623332 | 1.090558079 | 1.503813643 | 0.0025483 |
| **MMP7** | 1.117913049 | 1.039811329 | 1.201881101 | 0.002557498 |
| **ZBTB34** | 1.478606764 | 1.146552499 | 1.906827611 | 0.002579625 |
| **AQP9** | 0.904992134 | 0.848075625 | 0.965728454 | 0.002593681 |
| **RBMX** | 1.515742232 | 1.156304021 | 1.986912154 | 0.002599067 |
| **LINC01554** | 0.895921533 | 0.834040425 | 0.962393871 | 0.002615334 |
| **LIMS1** | 1.393621964 | 1.122294579 | 1.730545807 | 0.002661903 |
| **SERPINE2** | 1.185486487 | 1.060822283 | 1.324800802 | 0.00268646 |
| **PRPF40A** | 1.498853977 | 1.150658354 | 1.952415533 | 0.002696466 |
| **PLP2** | 1.195432113 | 1.063815543 | 1.343332447 | 0.002704999 |
| **SHKBP1** | 1.379038348 | 1.11765373 | 1.701552737 | 0.002723451 |
| **MOB1A** | 1.432862567 | 1.132279189 | 1.813241077 | 0.002752071 |
| **NRSN2** | 1.182047402 | 1.059446846 | 1.318835452 | 0.002757267 |
| **ITPR3** | 1.196968661 | 1.063957406 | 1.346608395 | 0.002776342 |
| **AL499602.1** | 1.216800134 | 1.069793219 | 1.38400818 | 0.002818058 |
| **GSTCD** | 1.445653623 | 1.134928824 | 1.84144975 | 0.002834925 |
| **EP400** | 1.491987106 | 1.146455716 | 1.941658533 | 0.002912392 |
| **OSBPL3** | 1.353345172 | 1.108818197 | 1.651797525 | 0.00292161 |
| **CIB2** | 1.262924881 | 1.082925891 | 1.472842481 | 0.002925682 |
| **MMP12** | 1.155003717 | 1.050404009 | 1.270019511 | 0.002927381 |
| **LOX** | 1.232398997 | 1.073848917 | 1.414358448 | 0.002939466 |
| **HTRA3** | 1.182377332 | 1.058768548 | 1.320417157 | 0.002943255 |
| **KDM5B** | 1.372970003 | 1.113621222 | 1.692717948 | 0.003002853 |
| **DBR1** | 1.481708119 | 1.142752477 | 1.92120253 | 0.003008949 |
| **ZNF107** | 1.403636083 | 1.121759681 | 1.756342545 | 0.00303119 |
| **NCKAP1** | 1.409334316 | 1.122734114 | 1.769094917 | 0.003096649 |
| **PLPP2** | 1.137269213 | 1.0443193 | 1.238492156 | 0.003108556 |
| **IGF2BP2** | 1.173224697 | 1.055295622 | 1.304332323 | 0.003119257 |
| **TMEM87B** | 1.385724334 | 1.116086123 | 1.720505155 | 0.003129585 |
| **ETNK1** | 1.485185555 | 1.142408529 | 1.930812032 | 0.003132534 |
| **PAK1** | 1.360044917 | 1.109049058 | 1.667845225 | 0.003133653 |
| **ZNF888** | 1.255179147 | 1.079307461 | 1.459708886 | 0.003168955 |
| **FNDC3B** | 1.354286319 | 1.107140141 | 1.656602778 | 0.003177794 |
| **AEBP2** | 1.512477483 | 1.148739939 | 1.991389051 | 0.003198846 |
| **PYCR1** | 1.15194356 | 1.04843627 | 1.265669649 | 0.003233543 |
| **PLAGL2** | 1.364373417 | 1.109267601 | 1.678147653 | 0.003262456 |
| **ATP2B1** | 1.34702978 | 1.104132129 | 1.643362403 | 0.003320651 |
| **TFRC** | 1.293677583 | 1.089051386 | 1.536751809 | 0.003378487 |
| **ZNF28** | 1.263429512 | 1.080426584 | 1.477429523 | 0.003401522 |
| **RNF44** | 1.371495323 | 1.110152213 | 1.694361726 | 0.003403026 |
| **CCL20** | 1.120461596 | 1.038301813 | 1.2091226 | 0.003418977 |
| **MADD** | 1.527740927 | 1.150011931 | 2.029537501 | 0.003449972 |
| **ZDHHC13** | 1.328781322 | 1.098198148 | 1.607778892 | 0.00346414 |
| **FAM98B** | 1.495069739 | 1.141671561 | 1.957860387 | 0.003467943 |
| **EHBP1L1** | 1.408246279 | 1.11913147 | 1.77205059 | 0.003500763 |
| **CD2AP** | 1.374616709 | 1.110214804 | 1.701986939 | 0.003508901 |
| **FNBP1L** | 1.311993239 | 1.092857631 | 1.575069077 | 0.003588034 |
| **UNC119B** | 1.340594587 | 1.10037453 | 1.633256493 | 0.003621607 |
| **SLC2A6** | 1.219644608 | 1.066694537 | 1.394525722 | 0.003680032 |
| **SERPINE1** | 1.146237865 | 1.045341237 | 1.256873064 | 0.003693711 |
| **MAP1S** | 1.473068353 | 1.134101425 | 1.913347718 | 0.003694591 |
| **LRRCC1** | 1.329318309 | 1.096727531 | 1.611236262 | 0.003721568 |
| **RTL6** | 1.272575842 | 1.081210768 | 1.497810901 | 0.003742807 |
| **PKDCC** | 1.216164706 | 1.065242907 | 1.388468848 | 0.003793077 |
| **KLHL42** | 1.406057392 | 1.115504196 | 1.772290411 | 0.003908416 |
| **TAF6** | 1.346250865 | 1.099896408 | 1.647783716 | 0.003934909 |
| **TEAD2** | 1.224965842 | 1.066890784 | 1.406461971 | 0.003996079 |
| **KIF5B** | 1.389785297 | 1.110607751 | 1.739140728 | 0.004016071 |
| **GNS** | 1.388325453 | 1.110066806 | 1.736334745 | 0.004041059 |
| **LINC00665** | 1.254436801 | 1.074729778 | 1.464192878 | 0.004059133 |
| **JRKL** | 1.388276163 | 1.109802109 | 1.736625556 | 0.004078553 |
| **TRIO** | 1.376445135 | 1.106687432 | 1.711956921 | 0.004094304 |
| **KCTD9** | 1.372932862 | 1.105686923 | 1.704772485 | 0.004110449 |
| **LZTS2** | 1.312114469 | 1.089745404 | 1.579859272 | 0.004142807 |
| **TWF1** | 1.417907843 | 1.11607366 | 1.801370933 | 0.004247615 |
| **DHX15** | 1.433999992 | 1.119839921 | 1.83629458 | 0.004275616 |
| **GTF3C4** | 1.496475187 | 1.134859539 | 1.973317322 | 0.004284971 |
| **APOC1** | 0.860055577 | 0.775427298 | 0.953919985 | 0.004336247 |
| **TCEAL9** | 1.194091964 | 1.056992545 | 1.348974147 | 0.004361919 |
| **UNC119** | 1.335038015 | 1.094156628 | 1.628950056 | 0.004422658 |
| **SFI1** | 1.369398425 | 1.102782252 | 1.700473545 | 0.004433797 |
| **USP33** | 1.451558102 | 1.122950781 | 1.876325265 | 0.004435272 |
| **AKR7A3** | 0.894010725 | 0.827562863 | 0.965793915 | 0.004466038 |
| **HDAC7** | 1.313858883 | 1.088450526 | 1.585947293 | 0.004475231 |
| **DCAF7** | 1.401576459 | 1.110295389 | 1.769273826 | 0.004508924 |
| **PURB** | 1.528923299 | 1.140307973 | 2.049978171 | 0.004547361 |
| **PTBP3** | 1.360658174 | 1.099839658 | 1.683327795 | 0.004561882 |
| **NFE2L3** | 1.246953667 | 1.070571211 | 1.452396097 | 0.004563625 |
| **MMP9** | 1.142962347 | 1.042083921 | 1.25360626 | 0.004591829 |
| **TOP1** | 1.468258864 | 1.125570179 | 1.915281812 | 0.004622122 |
| **UBE2E1** | 1.346809198 | 1.096016911 | 1.654988165 | 0.004625314 |
| **TTC36** | 0.862016898 | 0.777821243 | 0.955326353 | 0.004633031 |
| **BNIP3P11** | 1.367863963 | 1.101147278 | 1.699183984 | 0.004645499 |
| **SOCS5** | 1.488226155 | 1.129913509 | 1.960165155 | 0.004668229 |
| **CLSTN1** | 1.229723094 | 1.065066413 | 1.419835298 | 0.004810827 |
| **TNFRSF21** | 1.190332932 | 1.054519216 | 1.343638378 | 0.004820574 |
| **PPP1R18** | 1.26250246 | 1.073536256 | 1.484730909 | 0.004836528 |
| **PITX1** | 1.157002824 | 1.045212974 | 1.280749061 | 0.004909362 |
| **SLC6A6** | 1.197019473 | 1.056039332 | 1.356820316 | 0.004911264 |
| **FKBP9** | 1.349061551 | 1.094809049 | 1.662360272 | 0.004952596 |
| **GRAMD1A** | 1.246438437 | 1.068852505 | 1.453529621 | 0.004969088 |
| **C15orf39** | 1.309220488 | 1.084485777 | 1.580526294 | 0.005046392 |
| **SPTLC1** | 1.439403414 | 1.11546743 | 1.857411639 | 0.00511022 |
| **HKDC1** | 1.156111611 | 1.044448796 | 1.279712382 | 0.005123929 |
| **CD276** | 1.347864261 | 1.093137682 | 1.661948075 | 0.005218802 |
| **PPP5C** | 1.446185715 | 1.116156892 | 1.873798511 | 0.005247453 |
| **NAA35** | 1.47148274 | 1.121686103 | 1.930363091 | 0.005284879 |
| **SEPTIN7** | 1.48128694 | 1.12388937 | 1.952337175 | 0.005286837 |
| **KIF24** | 1.478585197 | 1.123177648 | 1.9464545 | 0.005301893 |
| **SLC6A1** | 0.851295866 | 0.76019179 | 0.953318178 | 0.005307131 |
| **ATP11A** | 1.292439578 | 1.078823582 | 1.54835331 | 0.005384526 |
| **SPRY4** | 1.313964796 | 1.084028814 | 1.592673056 | 0.00540181 |
| **ZNF83** | 1.207034679 | 1.057116221 | 1.378214322 | 0.005421935 |
| **SULT1C2** | 1.165789799 | 1.046282389 | 1.298947464 | 0.005438392 |
| **SPHK1** | 1.130426516 | 1.0367578 | 1.232557988 | 0.005470617 |
| **EIF4G2** | 1.388223117 | 1.101017474 | 1.750347719 | 0.005542215 |
| **GLIS2** | 1.209728597 | 1.057299297 | 1.384133406 | 0.005591496 |
| **BX571818.1** | 1.17396769 | 1.047842467 | 1.315274175 | 0.005677194 |
| **SUSD1** | 1.336394314 | 1.088119618 | 1.641317491 | 0.005686611 |
| **WSB2** | 1.395313879 | 1.10175707 | 1.767087205 | 0.005709097 |
| **ACTR2** | 1.381583763 | 1.098546962 | 1.737544009 | 0.005717783 |
| **PLTP** | 1.162961693 | 1.044817841 | 1.294464783 | 0.005743236 |
| **TMX2P1** | 1.317121342 | 1.082986851 | 1.60187414 | 0.005810378 |
| **PGAP4** | 1.194717821 | 1.052809374 | 1.355754144 | 0.005822081 |
| **HSPA13** | 1.332375179 | 1.086531993 | 1.633843853 | 0.00582591 |
| **FBH1** | 1.377364462 | 1.09695507 | 1.72945357 | 0.005838188 |
| **FUS** | 1.41458242 | 1.105047844 | 1.810820619 | 0.005909482 |
| **C3P1** | 0.891035613 | 0.820758369 | 0.967330329 | 0.005916553 |
| **PAK4** | 1.34736307 | 1.08925992 | 1.666624476 | 0.005996225 |
| **GTF2IRD1** | 1.355443034 | 1.090992798 | 1.683994453 | 0.006025217 |
| **PPARD** | 1.315751394 | 1.081580531 | 1.600622129 | 0.006065008 |
| **PAQR5** | 1.173497452 | 1.046721166 | 1.315628568 | 0.006092019 |
| **MED14** | 1.363321943 | 1.092286921 | 1.701610341 | 0.006134016 |
| **SWAP70** | 1.34210271 | 1.087069027 | 1.656969006 | 0.006212263 |
| **NUDT21** | 1.451405938 | 1.111152783 | 1.895850173 | 0.006270928 |
| **AC138207.3** | 1.231645388 | 1.060670696 | 1.430180325 | 0.006286902 |
| **PLEKHA2** | 1.312878907 | 1.079950247 | 1.596046696 | 0.006298418 |
| **KCTD10** | 1.440265521 | 1.108545624 | 1.871248892 | 0.006304634 |
| **PLIN3** | 1.28769067 | 1.07385568 | 1.544106246 | 0.00635209 |
| **ZMIZ2** | 1.416423991 | 1.102987279 | 1.818930246 | 0.006370102 |
| **PROSER1** | 1.390121202 | 1.097034722 | 1.761509385 | 0.006399896 |
| **SNX6** | 1.38995888 | 1.09688599 | 1.761336827 | 0.006422878 |
| **AL627309.7** | 1.244190984 | 1.06303627 | 1.456216733 | 0.006501314 |
| **SPATC1L** | 1.194879456 | 1.050858317 | 1.358638831 | 0.006588241 |
| **PELI1** | 1.26108821 | 1.066753656 | 1.490825427 | 0.006592503 |
| **GNA13** | 1.37371277 | 1.092466434 | 1.727363621 | 0.006595142 |
| **TRIP12** | 1.464968471 | 1.112104533 | 1.929793969 | 0.006614377 |
| **PLEKHG2** | 1.335136293 | 1.083673489 | 1.644950199 | 0.006633555 |
| **WSB1** | 1.301273442 | 1.075874208 | 1.573894568 | 0.006656687 |
| **TMEM132A** | 1.170585912 | 1.044669351 | 1.311679506 | 0.006676067 |
| **PHIP** | 1.402405742 | 1.09824167 | 1.790809728 | 0.006703371 |
| **FZD6** | 1.237868562 | 1.06089738 | 1.444360789 | 0.006708482 |
| **DYNC1I2** | 1.332899609 | 1.082792173 | 1.640777808 | 0.006725025 |
| **EZR** | 1.243330862 | 1.062081601 | 1.455511168 | 0.006744813 |
| **HGFAC** | 0.919496821 | 0.865314533 | 0.977071771 | 0.006758655 |
| **SLC12A2** | 1.20971947 | 1.054009097 | 1.388433173 | 0.006765113 |
| **MTPN** | 1.400220447 | 1.096602969 | 1.787900777 | 0.006945184 |
| **LAMA4** | 1.306776967 | 1.075691452 | 1.58750545 | 0.00704233 |
| **ZNF160** | 1.324659454 | 1.079475354 | 1.625532869 | 0.007096754 |
| **PSIP1** | 1.287283915 | 1.070953569 | 1.547312532 | 0.007140699 |
| **VGLL4** | 1.293335176 | 1.071969127 | 1.560414228 | 0.007241051 |
| **AC079466.1** | 1.117293414 | 1.030372479 | 1.21154689 | 0.007273627 |
| **SNN** | 1.365432664 | 1.087530316 | 1.714348861 | 0.007303807 |
| **FCGR2A** | 1.262181647 | 1.064646285 | 1.496367884 | 0.00733283 |
| **ZYX** | 1.326690773 | 1.078890685 | 1.631405695 | 0.007366887 |
| **MAP4K3** | 1.307300759 | 1.074628616 | 1.59034968 | 0.007367915 |
| **INPPL1** | 1.386165962 | 1.09130618 | 1.760693845 | 0.007450594 |
| **SLC66A3** | 1.287013113 | 1.069768904 | 1.548374369 | 0.007474342 |
| **ANKIB1** | 1.307717039 | 1.074085853 | 1.592166817 | 0.007546853 |
| **ABCC4** | 1.203592903 | 1.050417008 | 1.379105502 | 0.007626328 |
| **AL390728.6** | 1.245637228 | 1.059990355 | 1.463798324 | 0.007641829 |
| **MFSD6** | 1.236338587 | 1.057879851 | 1.444902368 | 0.007644005 |
| **AKR1B1** | 1.180991371 | 1.045096436 | 1.334556861 | 0.007649311 |
| **PRPF39** | 1.409526986 | 1.095207457 | 1.814054782 | 0.007666349 |
| **CDC42SE2** | 1.382194992 | 1.089467495 | 1.753575031 | 0.007683425 |
| **ZNF292** | 1.328699764 | 1.078135228 | 1.637496873 | 0.007685273 |
| **ATN1** | 1.336169768 | 1.079687345 | 1.65358023 | 0.007698286 |
| **BMF** | 1.209077126 | 1.051493355 | 1.390277446 | 0.007705869 |
| **MAP3K1** | 1.324912619 | 1.077098653 | 1.629742498 | 0.007746195 |
| **SLC37A1** | 1.318823963 | 1.075498287 | 1.617200758 | 0.007828023 |
| **TSPAN15** | 1.17101707 | 1.042387095 | 1.315519911 | 0.007832227 |
| **TUFT1** | 1.296287473 | 1.070566967 | 1.569599348 | 0.007848246 |
| **HMGN4** | 1.318146903 | 1.075076532 | 1.616174482 | 0.007906338 |
| **STK24** | 1.302977704 | 1.071670066 | 1.584210431 | 0.007951119 |
| **FABP4** | 0.869863511 | 0.784688379 | 0.964284101 | 0.008008905 |
| **RNF4** | 1.423288111 | 1.09558432 | 1.84901245 | 0.008200573 |
| **DRAM1** | 1.263357424 | 1.062253344 | 1.502534203 | 0.008225626 |
| **GMIP** | 1.293367553 | 1.068685602 | 1.56528695 | 0.008234999 |
| **TGFB1** | 1.181185969 | 1.043862406 | 1.336574903 | 0.00827277 |
| **ACSS1** | 1.212910654 | 1.050930999 | 1.39985618 | 0.008310553 |
| **ZDHHC20** | 1.317558547 | 1.073524954 | 1.617065834 | 0.008319192 |
| **NUP58** | 1.409976914 | 1.091954424 | 1.820620764 | 0.008425925 |
| **TCTN2** | 1.287482821 | 1.066801141 | 1.553815373 | 0.008437885 |
| **PIAS3** | 1.299813727 | 1.069094985 | 1.580323308 | 0.008536002 |
| **TES** | 1.224371536 | 1.052843839 | 1.423844261 | 0.008572193 |
| **SMARCA5** | 1.375816327 | 1.084494434 | 1.745394449 | 0.008585422 |
| **BAZ2A** | 1.314195139 | 1.071764529 | 1.611462981 | 0.008636836 |
| **CPVL** | 1.191647795 | 1.04538996 | 1.358368189 | 0.008680796 |
| **CYP3A4** | 0.93688034 | 0.892346958 | 0.983636201 | 0.00869098 |
| **ATP8B2** | 1.249731442 | 1.057969134 | 1.476251648 | 0.008716028 |
| **ATP1A1** | 1.166180852 | 1.039448573 | 1.308364661 | 0.008815722 |
| **C2CD5** | 1.353588378 | 1.079060374 | 1.697960135 | 0.008847096 |
| **TPM4** | 1.239281872 | 1.055272116 | 1.455377751 | 0.008897217 |
| **TMEM243** | 1.298197593 | 1.066739283 | 1.579877123 | 0.00919279 |
| **CNNM4** | 1.337127601 | 1.074420623 | 1.66406916 | 0.009237477 |
| **REST** | 1.430553893 | 1.092499591 | 1.873213003 | 0.009237562 |
| **IFFO2** | 1.302334502 | 1.067337141 | 1.589071614 | 0.009272935 |
| **ZDHHC23** | 1.342654039 | 1.075380392 | 1.67635553 | 0.009277428 |
| **WDR47** | 1.404973387 | 1.087472553 | 1.815172448 | 0.009279805 |
| **XPO7** | 1.344621954 | 1.075632998 | 1.680878333 | 0.009317348 |
| **HPR** | 0.904540498 | 0.838608197 | 0.97565647 | 0.009371796 |
| **ITGAM** | 1.219701307 | 1.049862617 | 1.417015191 | 0.009431787 |
| **DSC2** | 1.290277906 | 1.064144928 | 1.564464605 | 0.009531968 |
| **CBX6** | 1.220469636 | 1.049716952 | 1.418997882 | 0.009571254 |
| **PPP1R37** | 1.331329144 | 1.071814038 | 1.65367986 | 0.009685275 |
| **TUSC3** | 1.151223365 | 1.034713107 | 1.280852853 | 0.009687659 |
| **AC006329.1** | 0.850089124 | 0.751645094 | 0.961426509 | 0.009698528 |
| **UBR5** | 1.339646614 | 1.07310371 | 1.672394787 | 0.009786284 |
| **QKI** | 1.364947937 | 1.077821638 | 1.728563247 | 0.009826006 |
| **MAML1** | 1.364102097 | 1.077298329 | 1.727260202 | 0.009931245 |
| **SLC18B1** | 1.300028496 | 1.064674447 | 1.587409274 | 0.010024604 |
| **MGAT5** | 1.255563061 | 1.055690309 | 1.493277513 | 0.010094536 |
| **HNRNPUL1** | 1.385007097 | 1.080407208 | 1.775483027 | 0.010161964 |
| **CCNJ** | 1.275120551 | 1.059125754 | 1.535164652 | 0.010270417 |
| **CSRNP2** | 1.350074692 | 1.073161393 | 1.698441341 | 0.010381987 |
| **CYP8B1** | 0.929490582 | 0.878926866 | 0.982963174 | 0.010404771 |
| **MCUB** | 1.228634026 | 1.049271372 | 1.438656967 | 0.01054832 |
| **VEGFB** | 1.16412608 | 1.036117614 | 1.307949516 | 0.01055998 |
| **VEZT** | 1.332793855 | 1.069370593 | 1.661107452 | 0.010560173 |
| **UHRF2** | 1.403626276 | 1.082082622 | 1.82071746 | 0.010641632 |
| **CMTM3** | 1.22514904 | 1.047902386 | 1.432375945 | 0.010871273 |
| **SMARCAD1** | 1.334298577 | 1.068283082 | 1.666555169 | 0.011015836 |
| **SRC** | 1.194209811 | 1.041450828 | 1.36937533 | 0.011035793 |
| **MED23** | 1.353150597 | 1.071123511 | 1.709435486 | 0.011208573 |
| **MXRA7** | 1.245091522 | 1.050770905 | 1.475348139 | 0.011341489 |
| **SINHCAF** | 1.223397311 | 1.046465228 | 1.43024435 | 0.011412431 |
| **BLMH** | 1.206937083 | 1.043092629 | 1.396517512 | 0.011512538 |
| **IFNGR2** | 1.284336928 | 1.057723177 | 1.559501938 | 0.011518454 |
| **ACVR1** | 1.29340952 | 1.059076715 | 1.579591132 | 0.011643291 |
| **PACS1** | 1.291803118 | 1.058488075 | 1.576546147 | 0.011760675 |
| **FMNL2** | 1.193976581 | 1.040079509 | 1.370645286 | 0.011798355 |
| **TRAFD1** | 1.359115535 | 1.070092438 | 1.726201375 | 0.011892685 |
| **RHBDF2** | 1.254634057 | 1.051162971 | 1.497490551 | 0.011982703 |
| **ARHGAP18** | 1.305943787 | 1.060153602 | 1.608718935 | 0.012105991 |
| **MYLIP** | 1.259921477 | 1.051010373 | 1.510358194 | 0.012495936 |
| **TRIM47** | 1.209198112 | 1.041725727 | 1.40359409 | 0.012510661 |
| **PPM1M** | 1.285878867 | 1.055325918 | 1.566799823 | 0.012627346 |
| **ABCC10** | 1.327677887 | 1.062509816 | 1.659023328 | 0.012653915 |
| **RAI1** | 1.283559988 | 1.054753281 | 1.562001534 | 0.012698117 |
| **DUSP9** | 1.111485012 | 1.022752716 | 1.207915573 | 0.012775971 |
| **VCL** | 1.278340862 | 1.052885038 | 1.552073874 | 0.013118725 |
| **NACC1** | 1.390081653 | 1.071550973 | 1.803299189 | 0.013123342 |
| **HPD** | 0.930470865 | 0.878962329 | 0.98499788 | 0.013131113 |
| **SAMD4B** | 1.397189541 | 1.072583059 | 1.820034914 | 0.013160489 |
| **CLDN4** | 1.095638919 | 1.019153831 | 1.177864033 | 0.013367371 |
| **WWC3** | 1.318406943 | 1.0589633 | 1.641413698 | 0.013421761 |
| **FKBP10** | 1.146409415 | 1.02872036 | 1.277562491 | 0.01342403 |
| **ZNF550** | 1.305362416 | 1.056516639 | 1.612819879 | 0.013532895 |
| **ZDHHC21** | 1.424257803 | 1.075658407 | 1.885831297 | 0.013542321 |
| **CD7** | 1.160320316 | 1.031057084 | 1.30578923 | 0.013606407 |
| **POMK** | 1.330902061 | 1.060521297 | 1.67021662 | 0.013621254 |
| **ATXN2L** | 1.309906485 | 1.056867541 | 1.623528904 | 0.01370128 |
| **PCK1** | 0.918855248 | 0.859043601 | 0.982831331 | 0.013730464 |
| **GFPT1** | 1.319206402 | 1.05823165 | 1.644541185 | 0.013769672 |
| **RERE** | 1.321589878 | 1.058030531 | 1.650802841 | 0.014009197 |
| **MAP3K21** | 1.215489467 | 1.040224403 | 1.420284548 | 0.014035241 |
| **MICB** | 1.246970966 | 1.045471211 | 1.487306942 | 0.014109562 |
| **MBOAT1** | 1.172549143 | 1.032332357 | 1.331810907 | 0.014299136 |
| **ZMIZ1** | 1.262004264 | 1.04757907 | 1.520319379 | 0.014317909 |
| **NUFIP2** | 1.385328098 | 1.067230625 | 1.798237322 | 0.014332302 |
| **TRRAP** | 1.377856424 | 1.065815477 | 1.781254228 | 0.014426398 |
| **RCN2** | 1.300861695 | 1.053681485 | 1.606027222 | 0.014433448 |
| **RIF1** | 1.370613973 | 1.064644704 | 1.764516046 | 0.014446198 |
| **FAM118A** | 1.258471499 | 1.046634931 | 1.513183315 | 0.014499426 |
| **RAB8B** | 1.283369841 | 1.050623857 | 1.567676327 | 0.014538908 |
| **TSPAN3** | 1.285226275 | 1.050374346 | 1.572588463 | 0.014796415 |
| **GLYAT** | 0.918242046 | 0.85716383 | 0.983672461 | 0.015152152 |
| **NCEH1** | 1.166665957 | 1.03019612 | 1.321213921 | 0.015154351 |
| **CYB561** | 1.184309525 | 1.033183787 | 1.357540711 | 0.015154969 |
| **SRSF11** | 1.300977172 | 1.052076259 | 1.608763232 | 0.01516042 |
| **PRSS23** | 1.213241474 | 1.037692479 | 1.418488526 | 0.015353154 |
| **PRRC2C** | 1.282390782 | 1.048285674 | 1.568776678 | 0.015585234 |
| **PRDM4** | 1.363407781 | 1.060050638 | 1.753577339 | 0.015773117 |
| **RASEF** | 1.175015421 | 1.030715339 | 1.339517505 | 0.015843559 |
| **CRLF3** | 1.39291088 | 1.063762692 | 1.82390371 | 0.015980486 |
| **CLIC4** | 1.266749097 | 1.045033838 | 1.535503653 | 0.016010339 |
| **INIP** | 1.388210808 | 1.062817853 | 1.813226266 | 0.016082755 |
| **PRAME** | 1.10956359 | 1.01939715 | 1.20770532 | 0.016206691 |
| **RIDA** | 0.849858564 | 0.744268074 | 0.970429344 | 0.016242887 |
| **CRTC3** | 1.3795051 | 1.060884638 | 1.793818341 | 0.016347891 |
| **TBC1D1** | 1.313352277 | 1.051250641 | 1.640802046 | 0.016393658 |
| **CCNG2** | 1.294142811 | 1.047241345 | 1.599254674 | 0.016970802 |
| **MYO6** | 1.268884906 | 1.043052578 | 1.543612412 | 0.017242279 |
| **SOX9** | 1.129201149 | 1.021706011 | 1.248006004 | 0.017280324 |
| **ZNF92** | 1.344021413 | 1.053531266 | 1.714608402 | 0.017327901 |
| **ZNF496** | 1.234797661 | 1.037852945 | 1.469114936 | 0.017356442 |
| **MICAL1** | 1.218843117 | 1.035258731 | 1.434982868 | 0.017502261 |
| **TP53BP1** | 1.322893387 | 1.050120423 | 1.666520216 | 0.017546255 |
| **AOX1** | 0.915486502 | 0.851079155 | 0.984768021 | 0.017675289 |
| **TRAF5** | 1.288922448 | 1.044715704 | 1.590213557 | 0.017878678 |
| **ZNF468** | 1.199104684 | 1.031639785 | 1.393753967 | 0.017989907 |
| **CCDC14** | 1.290238912 | 1.044663748 | 1.593542854 | 0.018001373 |
| **VCAN** | 1.140358652 | 1.022766732 | 1.271470625 | 0.018011749 |
| **SCAF11** | 1.349125014 | 1.05145275 | 1.731069992 | 0.01855054 |
| **TMEM51** | 1.16326978 | 1.025543722 | 1.319491849 | 0.018658899 |
| **FNBP4** | 1.317718609 | 1.047004682 | 1.658428433 | 0.018700761 |
| **ABL1** | 1.295075944 | 1.043608829 | 1.607136365 | 0.018900464 |
| **SEMA4C** | 1.293955172 | 1.043361321 | 1.604736494 | 0.018952686 |
| **SALL2** | 1.194137061 | 1.029008319 | 1.385764619 | 0.019462738 |
| **AFTPH** | 1.357037008 | 1.050231698 | 1.753469683 | 0.01955558 |
| **PTGER4** | 1.244506156 | 1.035481615 | 1.49572484 | 0.019720638 |
| **SLCO4C1** | 1.149029223 | 1.022272714 | 1.291502881 | 0.019841681 |
| **GMCL1** | 1.283674439 | 1.040345754 | 1.583915789 | 0.019868877 |
| **TRAK2** | 1.312031982 | 1.043692223 | 1.649363561 | 0.020003735 |
| **LEMD3** | 1.349813088 | 1.047658998 | 1.739111081 | 0.020337399 |
| **AL354872.1** | 0.874071614 | 0.780069369 | 0.979401597 | 0.020422309 |
| **RAVER2** | 1.210812987 | 1.029976834 | 1.423399091 | 0.020457476 |
| **TIA1** | 1.28388068 | 1.039015141 | 1.586453879 | 0.020643035 |
| **ICAM1** | 1.152346363 | 1.021919837 | 1.299419087 | 0.020680417 |
| **AZGP1** | 0.900918814 | 0.824682555 | 0.984202594 | 0.020725583 |
| **UTRN** | 1.241164773 | 1.033400632 | 1.490699683 | 0.020806831 |
| **ACVR1B** | 1.286867221 | 1.038679604 | 1.594358105 | 0.021048286 |
| **LAMC1** | 1.194364205 | 1.026903455 | 1.389133368 | 0.021200064 |
| **ITGA2** | 1.187058148 | 1.025530358 | 1.37402763 | 0.021574797 |
| **BCL9L** | 1.20432053 | 1.027672458 | 1.411332889 | 0.021604453 |
| **ARL13B** | 1.325591122 | 1.04131939 | 1.687466726 | 0.022094593 |
| **CDC42EP1** | 1.210982047 | 1.02770754 | 1.426940508 | 0.022231376 |
| **OFD1** | 1.334014384 | 1.041797432 | 1.708196164 | 0.022338437 |
| **QPCT** | 1.149314336 | 1.019928496 | 1.295113773 | 0.022384113 |
| **HID1** | 1.166870761 | 1.021981272 | 1.332301689 | 0.02252521 |
| **ADNP2** | 1.306051548 | 1.038295741 | 1.642856247 | 0.022547896 |
| **NIBAN2** | 1.181565371 | 1.023612437 | 1.363891916 | 0.022683959 |
| **BPTF** | 1.291752865 | 1.036386914 | 1.610041039 | 0.022726658 |
| **PTP4A3** | 1.1460682 | 1.01921404 | 1.288710975 | 0.022729483 |
| **LOXL2** | 1.24448273 | 1.029947586 | 1.503704931 | 0.02347445 |
| **ARID5B** | 1.25840565 | 1.031477232 | 1.535259074 | 0.023486257 |
| **HMGN1** | 1.302112321 | 1.035909053 | 1.636723311 | 0.02367902 |
| **ELOVL7** | 1.12039805 | 1.015180133 | 1.236521234 | 0.023859029 |
| **ARAP1** | 1.364825605 | 1.041712146 | 1.788160903 | 0.024043332 |
| **CYP4A11** | 0.91110425 | 0.840287429 | 0.987889293 | 0.02412626 |
| **TLE3** | 1.26032171 | 1.030645331 | 1.541180816 | 0.024193956 |
| **UGCG** | 1.233052537 | 1.027529714 | 1.4796833 | 0.024329402 |
| **NBEAL2** | 1.276296326 | 1.031974731 | 1.578461432 | 0.024431316 |
| **COL9A2** | 1.151579288 | 1.01818047 | 1.3024556 | 0.024653536 |
| **YTHDC1** | 1.391846932 | 1.042449175 | 1.858352357 | 0.024971107 |
| **TMED3** | 1.16519008 | 1.019345722 | 1.33190133 | 0.025038786 |
| **RAPGEF1** | 1.326569038 | 1.036036633 | 1.698574508 | 0.025047735 |
| **SUGP2** | 1.315648001 | 1.034835988 | 1.67266087 | 0.025122817 |
| **GCC2** | 1.292002323 | 1.032393146 | 1.616893729 | 0.025187498 |
| **SMCHD1** | 1.29110766 | 1.032070935 | 1.61515932 | 0.025334995 |
| **CDS1** | 1.141741659 | 1.01632153 | 1.282639379 | 0.025571908 |
| **GAL3ST1** | 1.099888319 | 1.011675547 | 1.195792779 | 0.0256081 |
| **ZNF708** | 1.342727371 | 1.036558302 | 1.739329846 | 0.025622072 |
| **RGS1** | 1.130169528 | 1.014685096 | 1.258797598 | 0.026078177 |
| **BBX** | 1.279422154 | 1.029758378 | 1.589616636 | 0.02610005 |
| **SIPA1L3** | 1.226786085 | 1.024182412 | 1.469468798 | 0.026458158 |
| **ANKS1A** | 1.309231662 | 1.03191577 | 1.661073117 | 0.026509701 |
| **PLEKHB1** | 1.136714015 | 1.014997995 | 1.273025916 | 0.026582901 |
| **NCOR2** | 1.293877018 | 1.030220293 | 1.625009475 | 0.026688413 |
| **CCNE1** | 1.122411785 | 1.01333847 | 1.243225488 | 0.026828685 |
| **AC016735.1** | 1.117321641 | 1.012747365 | 1.232694048 | 0.026924861 |
| **MCAM** | 1.200746957 | 1.020783004 | 1.412438538 | 0.027226304 |
| **KANSL3** | 1.326630711 | 1.032099448 | 1.705212658 | 0.027339849 |
| **ETNPPL** | 0.908500708 | 0.834241204 | 0.989370379 | 0.0274128 |
| **CS** | 1.272481779 | 1.027151599 | 1.576407882 | 0.02744597 |
| **LINC01485** | 0.902536986 | 0.823842047 | 0.988749014 | 0.027591555 |
| **ITGB1** | 1.184675661 | 1.018838928 | 1.377505693 | 0.027627953 |
| **PMEPA1** | 1.125627917 | 1.013110451 | 1.250641731 | 0.027639284 |
| **RNF38** | 1.28370269 | 1.027819746 | 1.603289489 | 0.027673478 |
| **POFUT2** | 1.323564952 | 1.030661697 | 1.699708244 | 0.028047979 |
| **TGFBRAP1** | 1.348735365 | 1.03249286 | 1.761839868 | 0.028197847 |
| **HENMT1** | 1.179520764 | 1.017739142 | 1.36701948 | 0.02826601 |
| **CYP2C8** | 0.927619254 | 0.867367092 | 0.992056867 | 0.028328915 |
| **FLII** | 1.303022842 | 1.027788631 | 1.651962743 | 0.028788333 |
| **RAB31** | 1.198018891 | 1.018851479 | 1.408693311 | 0.028821045 |
| **TMEM263** | 1.314281987 | 1.02856573 | 1.679364858 | 0.028876568 |
| **RAVER1** | 1.231739679 | 1.021234023 | 1.485636596 | 0.029279622 |
| **POLR2A** | 1.271651452 | 1.024358119 | 1.578644602 | 0.029399657 |
| **CD83** | 1.24117993 | 1.021330544 | 1.508353615 | 0.029844171 |
| **ARMCX3** | 1.132195902 | 1.012158951 | 1.26646863 | 0.02990699 |
| **FLNA** | 1.146671567 | 1.013231188 | 1.29768576 | 0.030143577 |
| **DNM2** | 1.342819584 | 1.028630362 | 1.752976094 | 0.030194222 |
| **SKIL** | 1.240801248 | 1.020836999 | 1.508162163 | 0.030226947 |
| **ILDR1** | 1.166422143 | 1.01453507 | 1.341048384 | 0.030564629 |
| **COL16A1** | 1.16987195 | 1.014717007 | 1.348750805 | 0.030678897 |
| **ASPDH** | 0.911142151 | 0.837273362 | 0.991528045 | 0.030990552 |
| **CORO2A** | 1.193729049 | 1.016131219 | 1.40236715 | 0.031187108 |
| **SEL1L3** | 1.120440199 | 1.010300586 | 1.242586866 | 0.031234204 |
| **PPP1R12A** | 1.301954647 | 1.023997457 | 1.655361437 | 0.031279247 |
| **MAPK13** | 1.116406407 | 1.009791356 | 1.234278011 | 0.031536926 |
| **LGALS3BP** | 1.119057024 | 1.009907635 | 1.240003125 | 0.031694119 |
| **BLVRA** | 1.166056809 | 1.013456811 | 1.341634361 | 0.031813056 |
| **PRR12** | 1.249959035 | 1.019319993 | 1.532784209 | 0.032046013 |
| **GRK2** | 1.374018518 | 1.027427445 | 1.837528184 | 0.032160752 |
| **CERCAM** | 1.145815901 | 1.011609158 | 1.297827395 | 0.032228772 |
| **MAP4K5** | 1.303704267 | 1.022584908 | 1.662106298 | 0.032339227 |
| **MSI2** | 1.263792788 | 1.019845194 | 1.566092794 | 0.03239124 |
| **H2AW** | 1.104133536 | 1.008330939 | 1.209038438 | 0.032426218 |
| **STX2** | 1.26438783 | 1.019497917 | 1.568101864 | 0.032698053 |
| **LAMA5** | 1.176545039 | 1.013218171 | 1.366199569 | 0.032992263 |
| **FGD6** | 1.211911692 | 1.015312772 | 1.446578818 | 0.033317297 |
| **MAL2** | 1.148459916 | 1.010926312 | 1.304704569 | 0.033424783 |
| **RGS10** | 1.181032805 | 1.0130479 | 1.376873184 | 0.033539202 |
| **RPL39L** | 1.107091265 | 1.007882086 | 1.216065934 | 0.03368125 |
| **GALNT18** | 1.172580182 | 1.012002456 | 1.358637299 | 0.034112163 |
| **NEB** | 1.166857147 | 1.011511018 | 1.346061069 | 0.034261754 |
| **CAB39** | 1.345935891 | 1.022260342 | 1.77209596 | 0.034273882 |
| **ZNF146** | 1.2773718 | 1.018254594 | 1.602427059 | 0.034314789 |
| **BTN2A1** | 1.268130548 | 1.017574256 | 1.580381065 | 0.034422642 |
| **PPP1R9B** | 1.243814139 | 1.015179408 | 1.523941089 | 0.035262075 |
| **OSBPL8** | 1.261861481 | 1.01582286 | 1.567492188 | 0.035568064 |
| **PRRC2A** | 1.248831731 | 1.014925204 | 1.53664594 | 0.035731328 |
| **MED1** | 1.306893121 | 1.017618516 | 1.678398735 | 0.036012587 |
| **FAM3C2P** | 1.227940993 | 1.013235318 | 1.488143037 | 0.036255271 |
| **ADD3** | 1.174786414 | 1.00991703 | 1.366570795 | 0.036809825 |
| **PAPLN** | 1.129307007 | 1.007447236 | 1.26590681 | 0.036859104 |
| **MMGT1** | 1.263526045 | 1.01363471 | 1.575023083 | 0.037487498 |
| **CDC42SE1** | 1.23289585 | 1.012042157 | 1.501945514 | 0.037634099 |
| **FBLN1** | 1.118824022 | 1.006260881 | 1.243978788 | 0.037956131 |
| **B3GNT5** | 1.150592328 | 1.007594503 | 1.313884406 | 0.038293351 |
| **RP2** | 1.231684961 | 1.011173666 | 1.500284169 | 0.038418381 |
| **AP3M2** | 1.287938456 | 1.013026685 | 1.637454858 | 0.038864797 |
| **DCXR** | 0.882347863 | 0.783444447 | 0.993737021 | 0.039061309 |
| **APAF1** | 1.242310194 | 1.01093462 | 1.526641375 | 0.039075912 |
| **RELB** | 1.206012756 | 1.009391151 | 1.440934733 | 0.039121777 |
| **FBLIM1** | 1.157248554 | 1.007170899 | 1.32968915 | 0.0393232 |
| **MELTF** | 1.139853539 | 1.006300861 | 1.291130853 | 0.03951809 |
| **PXDN** | 1.185575021 | 1.008107314 | 1.39428423 | 0.039632115 |
| **RASA2** | 1.307418231 | 1.012200542 | 1.688738902 | 0.040088711 |
| **POSTN** | 1.104206743 | 1.004359629 | 1.213980028 | 0.040371849 |
| **PKN1** | 1.20896185 | 1.008284796 | 1.449579287 | 0.04045675 |
| **NSD3** | 1.275593239 | 1.009561502 | 1.611727576 | 0.041379663 |
| **HP** | 0.930939526 | 0.868878999 | 0.997432788 | 0.04205351 |
| **CYP4A22** | 0.908086631 | 0.827316055 | 0.99674281 | 0.042498233 |
| **PDX1** | 1.10728408 | 1.003359013 | 1.22197341 | 0.042698065 |
| **RECQL** | 1.196261403 | 1.005855256 | 1.422711007 | 0.042768082 |
| **TLNRD1** | 1.272818413 | 1.007593325 | 1.607857726 | 0.043030429 |
| **POLR2B** | 1.221829253 | 1.006062616 | 1.483870587 | 0.043286669 |
| **ERMP1** | 1.204388228 | 1.00546612 | 1.442665222 | 0.043471669 |
| **ARGLU1** | 1.249530197 | 1.006390854 | 1.551410873 | 0.043626329 |
| **ATP10D** | 1.242916106 | 1.00617359 | 1.535361752 | 0.043690189 |
| **NOTCH1** | 1.213130879 | 1.005471147 | 1.463678529 | 0.043703512 |
| **FOXJ1** | 1.115753745 | 1.002851399 | 1.241366787 | 0.044190958 |
| **IFT57** | 1.189224001 | 1.004471754 | 1.407957684 | 0.044245404 |
| **CYBB** | 1.145672228 | 1.003281634 | 1.308271586 | 0.044606281 |
| **LRIG3** | 1.177996252 | 1.00380855 | 1.38241019 | 0.044800233 |
| **MAP3K3** | 1.285479873 | 1.005463653 | 1.643479104 | 0.045130918 |
| **SMURF2** | 1.258171626 | 1.00468707 | 1.575610842 | 0.045424683 |
| **NIPA1** | 1.212072164 | 1.003696163 | 1.463708826 | 0.045684602 |
| **SUSD4** | 1.097588632 | 1.001686706 | 1.20267225 | 0.045924026 |
| **ITPRIPL2** | 1.209601893 | 1.003305685 | 1.458316007 | 0.046091361 |
| **ZNF14** | 1.201651223 | 1.003156033 | 1.439422796 | 0.046133343 |
| **PPM1H** | 1.151499545 | 1.002374717 | 1.322809904 | 0.046208596 |
| **ACTN4** | 1.253391914 | 1.003772716 | 1.565086663 | 0.046239965 |
| **TMEM181** | 1.252107242 | 1.003471375 | 1.562349046 | 0.04651975 |
| **PAQR8** | 1.171498577 | 1.002265606 | 1.369306607 | 0.046768265 |
| **COL4A2** | 1.158711492 | 1.001742783 | 1.340276512 | 0.04732177 |
| **NBPF15** | 1.184330766 | 1.001727022 | 1.40022115 | 0.047685478 |
| **ZNF664** | 1.248521942 | 1.002030682 | 1.555648012 | 0.047923918 |
| **P3H4** | 1.156264192 | 1.001102321 | 1.33547476 | 0.048273865 |
| **MIDEAS** | 1.273307648 | 1.001722791 | 1.618523987 | 0.048378658 |
| **CABIN1** | 1.283627724 | 1.001201178 | 1.645723328 | 0.048903436 |
| **BRD1** | 1.261523611 | 1.001049857 | 1.589772787 | 0.048969561 |
| **CYP2E1** | 0.954717042 | 0.911572074 | 0.999904073 | 0.049526631 |
| **PCGF2** | 1.224800091 | 1.000170384 | 1.499879709 | 0.049807664 |

HR, Hazard Ratio
